# FUS reads histone H3K36me3 to regulate alternative polyadenylation

**DOI:** 10.1093/nar/gkae184

**Published:** 2024-03-18

**Authors:** Junqi Jia, Haonan Fan, Xinyi Wan, Yuan Fang, Zhuoning Li, Yin Tang, Yanjun Zhang, Jun Huang, Dong Fang

**Affiliations:** Zhejiang Provincial Key Laboratory for Cancer Molecular Cell Biology, Life Sciences Institute, Zhejiang University, Hangzhou, Zhejiang 310058, China; Zhejiang Provincial Key Laboratory for Cancer Molecular Cell Biology, Life Sciences Institute, Zhejiang University, Hangzhou, Zhejiang 310058, China; Zhejiang Provincial Key Laboratory for Cancer Molecular Cell Biology, Life Sciences Institute, Zhejiang University, Hangzhou, Zhejiang 310058, China; Zhejiang Provincial Key Laboratory for Cancer Molecular Cell Biology, Life Sciences Institute, Zhejiang University, Hangzhou, Zhejiang 310058, China; Zhejiang Provincial Key Laboratory for Cancer Molecular Cell Biology, Life Sciences Institute, Zhejiang University, Hangzhou, Zhejiang 310058, China; Zhejiang Provincial Key Laboratory for Cancer Molecular Cell Biology, Life Sciences Institute, Zhejiang University, Hangzhou, Zhejiang 310058, China; Zhejiang Provincial Key Laboratory for Cancer Molecular Cell Biology, Life Sciences Institute, Zhejiang University, Hangzhou, Zhejiang 310058, China; Zhejiang Provincial Key Laboratory for Cancer Molecular Cell Biology, Life Sciences Institute, Zhejiang University, Hangzhou, Zhejiang 310058, China; Zhejiang Provincial Key Laboratory for Cancer Molecular Cell Biology, Life Sciences Institute, Zhejiang University, Hangzhou, Zhejiang 310058, China; Department of Medical Oncology, Key Laboratory of Cancer Prevention and Intervention, Ministry of Education, The Second Affiliated Hospital, Zhejiang University School of Medicine, Hangzhou, Zhejiang, China

## Abstract

Complex organisms generate differential gene expression through the same set of DNA sequences in distinct cells. The communication between chromatin and RNA regulates cellular behavior in tissues. However, little is known about how chromatin, especially histone modifications, regulates RNA polyadenylation. In this study, we found that FUS was recruited to chromatin by H3K36me3 at gene bodies. The H3K36me3 recognition of FUS was mediated by the proline residues in the ZNF domain. After these proline residues were mutated or H3K36me3 was abolished, FUS dissociated from chromatin and bound more to RNA, resulting in an increase in polyadenylation sites far from stop codons genome-wide. A proline mutation corresponding to a mutation in amyotrophic lateral sclerosis contributed to the hyperactivation of mitochondria and hyperdifferentiation in mouse embryonic stem cells. These findings reveal that FUS is an H3K36me3 reader protein that links chromatin-mediated alternative polyadenylation to human disease.

## Graphical Abstract

**Graphical Abstract:**
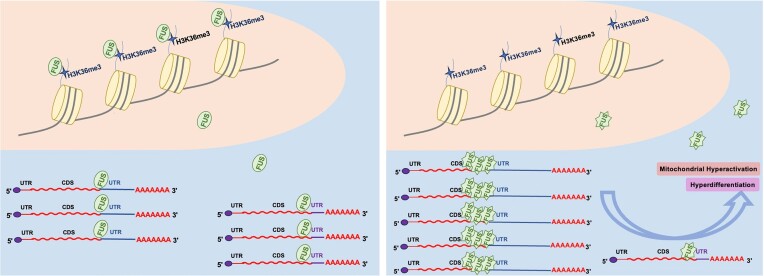

## Introduction

The cotranscriptional maturation of nascent RNA, involving capping, splicing, and 3′ terminal modification, is a key regulatory step in transcription (1–3). The 3′ termini of messenger RNA precursors (pre-mRNAs) are cleaved at the 3′ end, and a polyadenylated (poly(A)) tail is added downstream of the cleavage site (4,5). The poly(A) tail protects mRNA from enzymatic degradation and thus increases mRNA stability (6,7).

Cleavage and polyadenylation occur at polyadenylation sites that are located within introns, internal exons, or 3′ UTRs (3′ untranslated regions) (8,9). For many eukaryotic transcripts, alternative polyadenylation sites generate multiple transcript isoforms with different 3′ UTRs (10). This phenomenon, termed alternative polyadenylation sites (APA), allows a single gene to encode various mRNAs with different 3′ UTR contents (11–13). Found in > 60% of human genes, APA-induced 3′ UTR alterations in *cis-*regulatory elements, including protein-binding sites and regulatory RNA-binding sites, are involved in transcription and subsequently regulate mRNA stability, cellular RNA decay, protein diversification and translation efficiency (14–16). Dysregulation of APA is frequently found in the development and progression of a number of human diseases, including cancer and neurodegeneration (17).

Mutations in fused in sarcoma (FUS) protein have been implicated in amyotrophic lateral sclerosis (ALS) (18). The FUS protein has been identified as a major component in inclusion bodies generated in ALS (19,20). Interactions of FUS with the spliceosome (21,22), transcriptional machinery (23,24), and 3′-end-processing machinery have been identified (25,26). In addition, FUS can directly bind with mRNA and thus regulate pre-mRNA processing and mRNA polyadenylation (27). FUS enriched downstream of an APA increases the expression of alternative short transcripts and promotes polyadenylation by recruiting CPSF160. In contrast, FUS located upstream of an APA suppresses RNA polymerase II to downregulate the expression of alternative short transcripts and blocks polyadenylation (23). In line with these observations, genome-wide profiling reveals that the binding of FUS with mRNA is increased in exons that are undergoing alternative splicing (28). Depletion of FUS in neuronal cells changes the alternative splicing and polyadenylation of thousands of mRNAs that are enriched in neuronal development and functions (27,29). In mouse primary cortical neurons, the binding of FUS is scattered around alternatively spliced exons, including those associated with neurodegeneration, such as *Mapt*, *Camk2a* and *Fmr* (30). Recent studies also suggest that abnormal accumulation of pathological FUS aggregates linked to ALS disturbs protein homeostasis, leading to neurodegeneration. This is thought to occur through stress granule assembly, prion-like spreading and arginine methylation (31). In addition, FUS-dependent motor degeneration is caused by the toxic properties of ALS mutations rather than the loss of FUS function (32,33).

In eukaryotic cells, DNA is assembled into chromatin with nucleosomes, which consist of histones and 147 bp of DNA (34,35). Histone posttranslational modifications, called histone marks, have been reported to regulate chromatin structure and gene expression (36–38). Histone H3 can be trimethylated at lysine 36 (H3K36me3), which is a mark participating in several cellular processes (39–41). In gene body regions, H3K36me3 is deposited onto chromatin by SETD2 after transcription to mark actively transcribed genes (42). In promoters, SMYD5 catalyzes H3K36me3 deposition through its interaction with RNA polymerase II (43). H3K36me3 contributes to active gene regulation by serving as a mark of histone deacetylation to prevent spurious transcription in yeast (44,45). In human cells, H3K36me3 facilitates the recruitment of G9a and LSD2 to elongating chromatin. This maintains a repressive state via H3K9 methylation and H3K4 demethylation, which increases transcriptional fidelity and reduces cryptic transcription (46,47). ZMYND11 recognizes H3K36me3 and functions as a transcription corepressor (48–50). Most importantly, H3K36me3 has been proposed to regulate pre-mRNA splicing and APA because H3K36me3 is highly enriched at the 3′-end of gene bodies (51).

Studies on mechanisms causing APA selection have focused on mRNA cleavage/polyadenylation proteins or factors that regulate the localization of cleavage/polyadenylation complexes. In addition, chromatin structure and histone modification, such as H3K36me3, have been shown to regulate APA. However, the detailed molecular mechanism of APA regulation mediated via H3K36me3 has yet to be fully elucidated. In this study, we identified FUS as a reader of the H3K36me3 mark. Depletion of H3K36me3 at gene bodies released FUS from chromatin and increased the interaction of FUS with mRNA, which, in turn, led to APA selection occuring distant from the stop codon. The binding between FUS and H3K36me3 depends on proline residues in the ZNF domain of FUS, and therefore, mutations of proline residues cause loss of chromatin association and increased mRNA binding, resulting in an increase in distal APA selection. Moreover, loss of H3K36me3 recognition by FUS, which is mimicked by a proline mutation in FUS in ALS, contributes to hyperactivation of mitochondria and hyperdifferentiation in mouse embryonic stem cells (mESCs). Together, these observations led to the suggestion that FUS is an H3K36me3 reader protein that regulates APA in cells. These data expand our understanding of how H3K36me3-regulated APA affects mitochondrial function and cell differentiation and shed light on the causative effect of FUS mutations in ALS.

## Materials and methods

### Cell culture and cell lines

All mESCs were cultured in DMEM supplemented with 15% fetal bovine serum (FBS), 1% antibiotic solution (penicillin/ streptomycin), 1% glutamax, 1% MEM nonessential amino acids, 1% sodium pyruvate, 0.1 mM β-mercaptoethanol and 1000 U/ml recombinant leukemia inhibitory factor (LIF) on 0.1% gelatin-coated dishes. HEK293T, HeLa, and T/C28a2 cells were cultured in DMEM supplemented with 1% glutamax, 10% FBS and 1% antibiotic solution (penicillin/streptomycin). All cells were grown at 37°C and 5% CO_2_. mESCs and HEK293T cells were purchased from ATCC. T/C28a2 cell, which was the immortalized human juvenile chondrocyte, was a kind gift from Dr. Jennifer J. Westendorf.

*Fus* KO cell lines were constructed by two independent sgRNAs, which were cloned into pSpCas9(BB)-2A-Puro from Feng Zhang (Addgene plasmid #48139). *Fus* mutant cell lines, including FUS-P421A and FUS-P423L, were constructed by a sgRNA plasmid and single-strand DNA (Supplemental Table S2), which were co-transfected into WT mESCs. Cells were grown under puromycin selection (2 μg/ml) for 48h and then around 100 single clones were analyzed to identify the correct cell lines. Individual clones were picked under the microscope for the following genotyping.

### Primers

Primers used in this study were listed in Supplemental Table S2.

### Cell proliferation assay and alkaline phosphatase staining

Five hundred mESCs were seeded into each well of a 96-well plate and were cultured in growth medium. Cell Titer-Blue kit (Promega, Cat. #G8081) was used to measure cell viability at 24, 48 72 and 96 h, respectively. The fluorometric signals were recorded by microplate reader (BioTek, Synergy NEO2).

For alkaline phosphatase staining, mESCs were plated in a 24-well plate and were then fixed with 1% paraformaldehyde. INT/BCIP (Brown) kit (Sangon Biotech, Cat. #C500033) was used to detect the activity of alkaline phosphatases in mESCs under the manufacturer's instruction. T/C28a2 cells were used as the negative controls.

### Protein purification

The WT and mutant FUS were cloned into the GST-tagged pGEX-6p-2 vector. Briefly, the plasmids were transformed into *E.coli* BL21 cells (Weidi Biotechnology) and grew to OD_600_ at around 0.6. Isopropyl β-d-1-thiogalactopyranoside (IPTG) was added to a final concentration at 0.2 mM to induce the expression of recombinant proteins. The lysates were bound to GST-Seflnose Resin beads (Sangon Biotech, Cat. #C600031) at 4°C for 2–4 h. The beads were extensively washed and stored in PBS before usage.

### Reconstitution of octamer and nucleosome

The reconstitution of octamer and nucleosome was performed before (43). In brief, the plasmids pRSFDuet1-hH3.3/H4 and pRSFDuet1-hH2A/H2B, which were kind gifts from Dr Ruiming Xu, were transformed together into *Escherichia coli* BL21 cells (Weidi Biotechnology). Cells were grown to an OD_600_ at around 0.6 until 0.2 mM IPTG was added to induce the expression of histone proteins. After purified through HiTrap Heparin HP column (GE Healthcare) with a gradient of 0.5–2 M NaCl buffer (diluted in 20 mM Tris pH 8.0, 10 mM β-mercaptoethanol and 1 mM PMSF), the fractions with octamers were collected and dialyzed to histone gel filtration buffer (2 M NaCl, 20 mM Tris pH 8.0, and 10 mM β-mercaptoethanol). The assembled histone octamers were further purified on a HiLoad Superdex 200 gel filtration column in histone gel filtration buffer. The purity of proteins was checked before sample collection.

H3Kc36me3 octamer was reconstituted as described before (52). In brief, the K36 in human H3.3 was mutated to cysteine and the C110 was mutated to alanine. A methyl-lysine analog is generated by incubating the reconstituted H3Kc36 octamer with (2-bromoethyl) trimethylammonium bromide. An acetyl-lysine analog was generated by first incubating the reconstituted H3K_c_36 octamer with 0.2 M sodium acetate pH 4.0 followed by adding 50 mM *N*-vinyl-acetamide (Macklin, Cat. # 5202-78-8), 5 mM VA-044 (Macklin, Cat. # 27776-21-2) and 15 mM GSH (Macklin, Cat. # R917465) in sequence. The sample then was incubated in a 365 nM ultraviolet cross-linker machine for 2 h.

For nucleosome reconstitution, the purified histone octamers were titrated with ‘601’ DNA as previously described at a molar ratio of 0.9:1.0 in initiation buffer (2 M KCl, 10 mM Tris pH 7.5, 1 mM EDTA and 1 mM DTT) (53). After dialyzing step-wisely with final buffer (0.2 M KCl, 10 mM Tris pH 7.5, 1 mM EDTA and 1 mM DTT) at 4°C for 36 h, the samples were concentrated to at least 1 ml and further loaded to a HiLoad Superdex 200 gel filtration column with reconstitution buffer (25 mM Tris pH 7.5, 1 mM EDTA and 10% glycerol). The first fraction was collected as reconstituted nucleosomes. The quality of nucleosomes was checked by native gel with Coomassie brilliant blue (CBB) staining and DNA staining. The assembled octamers and reconstituted nucleosomes were aliquoted to around 20 μl each and stored at –80°C before usage.

For the assay of H3K36_c_me3 and H3K36me3 antibody competitive binding with FUS. FUS was incubated with H3K36_c_me3 in TBS at 4°C for 2 h. Antibodies were added into the incubated sample with different concentrations and further incubated at 4°C for 2 h. The pre-washed GST Seflnose Resin beads (Sangon Biotech, #C600031) were then added and rotated at 4°C for 1 h in a rotisserie-style tube rotator. The beads were extensively washed by TBS and boiled with SDS loading buffer before analysis.

### Separation of cell fractionation

4 × 10^6^ cells were collected and resuspended with 200 μl extraction buffer (20 mM HEPES pH 7.5, 50 mM NaCl, 3 mM MgCl_2_, 0.3 M sucrose and 0.5% Triton X-100). 100 μl sample was saved as whole cell extraction. The other 100 μl sample was incubated on ice for 10 min and centrifuged at 13 000 rpm, 4°C, for 10 min. The supernatant was saved as the cytoplasmic fraction. The nuclear pellets were washed with extraction buffer once and saved as the nuclear fraction. All samples were boiled in SDS loading buffer and then analyzed by western blotting.

### Immunoprecipitation (IP)

Human WT *Fus* was cloned into FLAG-tagged pcDNA3.1 vector to be overexpressed in HEK293T cells for IP. The empty vector and WT FUS transfected HEK293T cells were lysed in IP lysis buffer (50 mM HEPES pH 7.4, 200 mM NaCl, 0.5% NP-40, 10% glycerol and 1 mM EDTA), and then homogenized by a glass Dounce homogenizer with 50 passages. After being rotated at 4°C for 30 min and spun at 13 000 rpm at 4°C for 30 min, the collected supernatants were used to bind with Anti-DYKDDDDK G1 Affinity Resin beads (GenScript, Cat. #L00432) at 4°C overnight. The beads were extensively washed with washing buffer (50 mM HEPES pH 7.4, 100 mM NaCl, 0.01% NP-40, 10% glycerol and 1 mM EDTA). Bead-bound proteins were eluted by boiling with SDS loading buffer and then subjected to western blotting analysis.

### Mono-nucleosome IP

Human WT and mutant FUS were cloned into FLAG-tagged pcDNA3.1 vector to be overexpressed in HEK293T cells for mono-nucleosome IP. The harvested cells were resuspended in 10 ml PBS at room temperature and then fixed with 0.27 ml of freshly prepared 37% paraformaldehyde (PFA) for 3 min. Samples were mixed with 0.5 ml 2.5 M glycine and incubated at room temperature for 5 min. After washing with 5 ml 1× cold TBS twice, cells were lysed in cell lysis buffer (10 mM Tris pH7.5, 10 mM NaCl, 0.5% NP-40 and 1 mM PMSF) and incubated on ice for 10 min. The lysates were centrifuged at 3000 rpm and 4°C for 5 min and then washed with MNase digestion buffer (20 mM Tris pH 7.5, 15 mM NaCl, 60 mM KCl, 1 mM CaCl_2_ and 1 mM PMSF). After being centrifuged at 3000 rpm at 4°C for 5 min, the pellets were resuspended in 500 μl MNase digestion buffer with 0.5 μl MNase (NEB, M0247S) and incubated at 37°C, 1000 rpm for 20 min to digest chromatins into mono-nucleosomes. The reaction was stopped with 500 μl of 2× Stop/ChIP buffer (100 mM Tris pH8.1, 20 mM EDTA, 200 mM NaCl, 2% Triton X-100, 0.2% sodium deoxycholate and 1 mM PMSF). Then 100 ug/ml RNase A was added as indicated to the sample to digest RNA at 37°C for 30 min and the samples were centrifuged twice at 15 000 rpm and 4°C for 10 min. The supernatants were transferred to 30 μl Anti-DYKDDDDK G1 Affinity Resin beads, which were pre-washed with 1× Stop/ChIP buffer twice and rotated at 4°C overnight. The beads were extensively washed with 1X Stop/ChIP buffer five times. Beads-bound proteins were eluted by boiling with 50 μl SDS loading buffer and then subjected to Western blotting analysis.

As for *Setd2*/*Nsd1* knockdown HEK293T cells, WT and mutant FUS plasmids were first transfected, respectively. After 24 h, the WT and mutant FUS overexpressed cells were infected with viruses generated from shRNA. After another 72 h, cells were harvested for IP assay.

### Immunofluorescence

Cells that grew on coverslips in a 24-well plate were washed with 1 ml PBS once and then fixed with 500 μl 4% PFA for 10 min. After being washed with 1 ml PBS twice, cells were permeabilized with 0.5% Triton X-100 solution (20 mM HEPES pH 7.5, 50 mM NaCl, 3 mM MgCl_2_ and 0.3 M sucrose) with 5% normal goat serum for 1 h at room temperature. Cells were washed with PBS twice. Primary antibodies were diluted at 1:100 in PBS-T (1× PBS with 0.1% Tween 20) to be incubated with cells at 4°C overnight. After being washed with 1 ml PBS-T 3 times, cells were incubated with Alexa Fluor 488-conjugated antibodies (1:1000 dilution in PBS-T) for 1 h in the dark. Cells were then washed with 1 ml PBS-T 3 times in the dark. DAPI (Invitrogen, Cat. #D1306) was diluted at 1:1000 in PBS and added to cells for 5 min in the dark. After being washed with PBS twice in the dark, slides with Antifade reagents (Biosharp, Cat. #BL701A) were sealed with mounting medium and examined under a fluorescence microscope (Zeiss, LSM 880).

### Microscale thermophoresis (MST)

Protein–RNA interactions were analyzed by MST. RNAs were synthesized in the Sequencing Company (YouKang) modified with Cy5 at 3′ terminal, which was dissolved in MST buffer (PBS containing 0.05% BSA and 0.05% Tween-20) with a concentration of 5 nM. The purified WT FUS and mutants were also dissolved in MST buffer. Assays were performed with the NT.115 Monolith instrument (Nano Temper Technologies) using a red LED for excitation in three independent replicates. RNA sequences used: 5XGGUG-3'Cy5, 5′-GGUGGGUGGGUGGGUGGGUG-cy5-3′, 4XUUAGGG-3'Cy5: 5-UUAGGGUUAGGGUUAGGGUUAGGG-cy5-3′.

### Embryoid body formation

mESCs in good condition were diluted to 10 000 cells/ml in growth medium without LIF and then dropped 30 μl/drop (300 cells/drop) on the lid of a 15 cm-Petri dish. Around 120 drops were generated per lid. Cells were incubated at 37°C with 5% CO_2_ for three days and then transferred into the low-attachment dish with 10 ml growth medium lacking LIF. The medium was changed every 2 days. Samples were collected at 0, 3 and 5 days.

### RT-qPCR

RNA was extracted by the UNIQ-10 column total RNA purification kit (Sangon Biotech, Cat. #B511361-0100). 500 ng RNA was used for reverse transcription by HiScript® III RT SuperMix for qPCR (+gDNA wiper) (Vazyme, Cat. #R323-01) following the manufacturer's instructions. As for qPCR, two-step (95°C 30 s; 95°C 5 s and 60°C 15 s for 40 cycles) PCR was performed in a 10 μl reaction with 0.1 μl 10 mM primers and 5 μl Hieff® qPCR SYBR® Green Master Mix (YEASEN, Cat. #11201ES08) by using Quantagene q225 qPCR system (Kubo Technology, Beijing). β-actin was used as a control to normalize the expression of other target genes. Primers used were listed in Supplemental Table S2.

For different polyadenylation detection, ‘RT-primer’ was used for RT and ‘APA-check-R’ was used for qPCR to detect different APA sites. Because the qPCR results with the proximal APA site contained all the RNA and results with the distal APA site contained only distal APA, we compared the ratio of distal APA to all APA.

For RNA stability, mESCs were treated with 4 μg/ml of actinomycin D (Sigma-Aldrich). Cells were collected at 3.5 h after treatment of actinomycin D (0 and 3.5 h). Samples were collected and RNA was extracted for RT-qPCR. Total RNA before and after the treatment of actinomycin D for 3.5 h was collected. The same amounts of human RNA were spiked in before the reverse transcription. Gene expressions were normalized to human *Actin* and the expression levels before actinomycin D treatment were further normalized as 1. The relative mRNA levels were then calculated as compared to the levels before actinomycin D treatment, respectively.

### Reactive oxygen species (ROS) assay

20 000 mESCs were seeded into each well of a 96-well plate. The ROS of cells was measured by the reactive oxygen species assay kit (Beyotime, Cat. #S0033S) following the manufacturer's instructions. DCFH-DA (10 mM) was diluted at 1:1000 in the medium without fetal bovine serum (FBS). 100 μl diluted DCFH-DA was added into each well of the 96-well plate. Cells were incubated at 37°C with 5% CO_2_ for 30 min and then washed with medium without FBS three times. The fluorometric signals were quantified by a microplate reader (BioTek, Synergy NEO2) at 488/525 nm of excitation and emission.

### Seahorse XF-24 metabolic flux analysis

Mitochondrial respiration was characterized as an indicator of cellular metabolism by extracellular flux analysis using Agilent Seahorse XF24 Flux Analyzer (Agilent Seahorse Bioscience). For this purpose, the oxygen consumption rate (OCR) was measured according to the manufacturer's protocol in the function of time and added respiration modulators. The cells were cultured overnight on XF-24 plates at a density of 4 × 10^4^ cells/well. The medium was replaced with Seahorse XF assay medium (Seahorse Bioscience) in a non-CO_2_ incubator at 37°C for 1 h before measurement. The basal OCR was determined before the addition of modulators, followed by the sequential injections of oligomycin (ATP synthase inhibitor, 1 μM), carbonyl-cyanide-4-(trifluoromethoxy) phenyhydrazone (FCCP, an uncoupling agent of mitochondrial respiration to allow maximum electron transport, 0.3 μM), and a mix of rotenone and antimycin A (RA, Complex I/III inhibitor, respectively, 0.5 μM). Four mitochondrial respiration parameters were determined: basal, ATP production-linked, maximal and respiratory capacity. Each measurement was averaged from triplicate wells.

### Antibodies

Nuclear receptor binding SET domain protein 1 (NSD1) antibody (Abbexa, Cat. #135901); rabbit monoclonal [EPR18656] to C14orf169/NO66 (Abcam, Cat. #ab192861); rabbit polyclonal anti-histone H3 (Abcam, Cat. #ab1791); rabbit monoclonal anti-histone H3K27me3 (Cell Signaling Technology, Cat. #9733); rabbit monoclonal anti-H3K36me2 (Cell Signaling Technology, Cat. #2901); rabbit polyclonal anti-H3K36me3 (active motif, Cat. #61101); rabbit polyclonal anti-histone H3K36me3 (Cell Signaling Technology, Cat. #4909); rabbit polyclonal anti-histone H3K36me3 (Abcam, Cat. #ab9050); mouse monoclonal anti-β-tubulin (Cell Signaling Technology, Cat. #86298); rabbit monoclonal anti-SETD2 (Abcam, Cat. #ab239350); rabbit monoclonal anti-EZH2 (Cell Signaling Technology, Cat. # 5246); mouse monoclonal anti-FLAG (GenScript, Cat. #A00187); rabbit polyclonal anti-FLAG Tag (Sangon Biotech, Cat. #D110005); rabbit polyclonal anti-GST Tag (Sangon Biotech, Cat. #D110271); mouse monoclonal anti-FUS/FLS (Santa Cruz BiotechnologyInc, Cat. #47711); peroxidase AffiniPure goat anti-rabbit IgG (H + L) (Jackson ImmunoResearch Laboratories, Cat. #111-035-003, lot 158673); peroxidase AffiniPure goat anti-mouse IgG (H + L) (Jackson ImmunoResearch Laboratories, Cat. #115-035-003, lot 158673); Alexa Fluor® 594 AffiniPure Alpaca Anti-Mouse IgG (H + L) (Jackson ImmunoResearch Laboratories, Cat. # 615-585-214).

### CUT&Tag

CUT&Tag was performed as described before (43,54). In brief, 10^5^ cells were collected in NE buffer (20 mM HEPES pH 7.5, 0.5 mM spermidine, 10 mM KCl, 0.1% TritonX-100, 10% glycerol and 1 mM PMSF) and kept on ice for 10 min. 10 μl ConA beads which were pre-washed by binding buffer (20 mM HEPES pH 7.5, 10 mM KCl, 1 mM CaCl_2_ and 1 mM MnCl_2_) were added to each sample and incubated at room temperature for 10 min. After being washed with washing buffer (20 mM HEPES pH 7.5, 0.5 mM spermidine, 150 mM NaCl and 0.1% BSA) once, beads were incubated with blocking buffer (20 mM HEPES pH 7.5, 0.5 mM spermidine, 150 mM NaCl, 0.1% BSA and 2 mM EDTA) at room temperature for 5 min. Samples were incubated with diluted primary antibodies (1:100 dilution in washing buffer) at room temperature for 2 h. After washing with washing buffer once, secondary antibodies were added at a 1:100 dilution and incubated at room temperature for 30 min. PA-Tn5 transposons were then added and incubated at room temperature for 30 min. Beads were washed with washing buffer twice and incubated with 10 mM MgCl_2_ in washing buffer at 37°C for 1 h. The 30 μl tagmentation reaction was stopped by adding 2.25 μl 0.5 M EDTA, 2.75 μl 10% SDS and 0.5 μl 20 mg/ml Proteinase K and incubated at 55°C for 30 min, and then at 70°C for 20 min. DNA was purified by 0.9× of VAHTS DNA clean beads (Vazyme, Cat. #N411-03).

Sequencing raw reads were trimmed to remove adapters and low-quality sequences using Trim Galore (version 0.6.6) (https://www.bioinformatics.babraham.ac.uk/projects/trim_galore/) with the parameters ‘-q 20 –paired’. Then trimmed reads were uniquely mapped to the mouse reference genome (mm9) using Bowtie2 (55) (version 2.4.2). The output SAM files were converted to sorted BAM files using SAMtools (56) (version 1.11). Peaks were identified using MACS2 (57) (version 2.2.7.1) with the parameters ‘–nomodel -f BAMPE -g mm’. For H3K27me3 and H3K36me3 modifications, parameter ‘–broad’ was specified to identify broad peaks. Genome coverage bigwig files were generated by BEDtools (58) (version 3.5.0) genomecov and bedGraphToBigWig (version 4) (https://www.encodeproject.org/software/bedgraphtobigwig/) with the parameter ‘-scale 10000000/mapped_reads_number’. Gene Ontology (GO) analysis was performed by clusterProfiler (59) (version 4.2.2). The Venn plots were plotted by R package VennDiagram (version 1.7.1) (https://github.com/cran/VennDiagram). A 1 kb sliding window across the whole genome was used to calculate the Pearson product moment correlation.

### RIP-seq

RIP-seq was conducted as described before (60). In brief, cells were collected and washed with cold PBS once. HeLa cells were spiked in as the internal control for normalization. 1 ml cold polysome lysis buffer (50 mM KCl, 25 mM Tris pH 7.4, 5 mM EDTA, 0.5% NP-40, 0.5 mM DTT, 100 U/ml RNAase inhibitor and 1× protease inhibitor cocktail) was added and incubated on ice for 10 min. Samples were cleaned by centrifugation at 14 000 rpm, 4°C for 10 min. 10% of the lysate was saved as the input sample. Others were incubated with Anti-DYKDDDDK G1 Affinity Resin beads (GenScript, Cat. #L00432) which were washed with NT2 buffer (50 mM Tris pH 7.4, 150 mM NaCl, 1 mM MgCl_2_ and 0.05% NP-40) at 4°C for 4h. Beads were washed with NT2 buffer 5 times to remove unbound material. RNA was purified by Trizol reagent (Sangon Biotech, Cat. #B610409) and subjected to library preparation by RNA-seq library prep kit (VAHTS, Cat. #NR604) along with the input samples.

Sequencing raw read trimming, mapping, and peak calling were performed as the CUT&Tag analysis. PCR duplicates were removed using MarkDuplicates tool of Picard (version 2.23.3) (https://broadinstitute.github.io/picard/) with the parameter ‘–REMOVE DUPLICATES = true’. Genome coverage bigwig files were generated by deepTools (61) (version 3.5.0) bamCoverage with the parameter ‘-scaleFactor 1 000 000/(the number of reads mapped to hg19 genome)’. Aggregation plots were generated by deepTools (58) (version 3.5.0) computeMatrix and plotProfile. A 10 bases sliding window across the whole genome was used to calculate the Pearson product moment correlation.

### FUS RIP qPCR in the nucleus/cytoplasm

Freshly cultured cells were collected and washed with cold PBS twice. 100 μl cold NE buffer (20 mM Tris–HCl pH 7.5, 10 mM KCl, 0.5 mM spermidine, 0.1% Triton X-100, 1 mM PMSF, 100 U/ml RNAase inhibitor) was added to separate the nucleus and cytoplasm. 1 ml cold polysome lysis buffer was added and incubated on ice for 10 min. 10% of the lysate was taken as input, and others were incubated with Anti-FUS antibodies coated beads slurry. Beads were washed with NT2 buffer 5 times to remove unbound material. RNA was purified by Trizol reagent (Sangon Biotech, Cat. #B610409) for RT-qPCR.

### Bowl-seq

Binding oligo: /Index//iSp18/ACTCTGCGTTGATACCACTGCT/iBiodT/CCG/iSpC3/CGGAAGCAGTGGTATCAACGCAGAGTNNNNNNNNNNNN/Reverse complementary sequence of index//ideoxyU/TTTTTTTT/3InvdT/ was synthesized for the enrichment and ligation of poly(A) mRNA. /Index/ was designed as the 8 bp of sample index (Supplemental Table S3). /iSp18/ was synthesized as an internal 18-atom hexaethyleneglycol spacer. /iBiodT/ was biotin labeled dT. /iSpC3/ was a C3 Spacer designed to increase oligos’ self-anneal. NNNNNNNNNNNN was 12 N as the UMI (unique molecular identifiers) for each sequencing read. /ideoxyU/ was used for USER enzyme digestion to remove the following oligo dT before sequencing. /3InvdT/ was 3′ inverted dT which blocked the reverse transcription from undigested oligo. Low salt buffer (0.15 M NaCl, 20 mM Tris pH 7.5 and 1 mM EDTA) was prewarmed at 70°C. Binding oligos were dissolved in Low salt buffer to make a final concentration of 8 μM. 10 μl of binding oligo was added to 20 μl Streptavidin Magnetic Beads (Thermo, Cat. #65001) which were washed with Washing/binding buffer (0.5 M NaCl, 20 mM Tris-pH 7.5 and 1 mM EDTA) twice. Samples were incubated at room temperature for 30 min with occasional agitation by hands. Beads were then washed with Washing/binding buffer twice. 5 μg total RNA, which was extracted by Trizol reagent (Sangon Biotech, Cat. #B610409) following the manufacturer's instructions, was dissolved in 50 μl of Washing/binding buffer, denatured at 65°C for 5 min, and quickly chilled on ice for 2 min, before being incubated with 20 μl of beads. Samples were incubated at room temperature for 30 min and then washed with Washing/binding buffer twice. 0.8 μl 10 mM ATP, 4 μl T4 RNA ligase Reaction buffer (NEB, Cat. # B0216S), 8 μl 50% PEG8000, 1 μl T4 RNA ligase 2 (NEB, Cat. # M0239), 2 μl RNase inhibitor (NEB, Cat. # M0314S) and 4.2 μl H_2_O were added and incubated at room temperature for 2 h. Beads were washed with Washing/binding buffer twice, followed by 1× CutSmart buffer (NEB, Cat. # B7204) once. 49 μl 1× CutSmart buffer and 1 μl USER enzyme (NEB, Cat. # M5507) were mixed with the beads and incubated at 37°C for 30 min. After being washed with pre-warmed 70°C Low-salt buffer at room temperature for 2 min, beads were washed with H_2_O once. RT reaction was conducted by adding 4 μl 5× SuperScript IV RT Reaction Buffer (Thermo, Cat. #18091200), 1 μl 10 mM DTT, 1 μl 10 mM dNTP, 1 μl RNase inhibitor, 1 μl TSO oligo AAGCAGTGGTATCAACGCAGAGTACATrGrGrG. 3 μl 50% PEG8000, 1 μl SuperScript IV Reverse Transcriptase (Thermo, Cat. #18091200), and 8 μl H_2_O and incubating at 50°C for 30 min. Beads were then washed with low salt buffer and subsequent H_2_O once. Pre-PCR was conducted by using 25 μl NEBNext^®^ High-Fidelity 2× PCR Master Mix (NEB, Cat. #M0541S), 1 μl 20 μM PCR oligo AAGCAGTGGTATCAACGCAGAGT, and 24 μl H_2_O at 98°C for 3 min; 10 cycles of 98°C for 10 s, 60°C for 15 s, and 72°C for 3 min; 72°C for 5 min. Samples were purified by 0.9× of VAHTS DNA clean beads to 20 μl. The purified sample was used to construct the sequencing library for Sequel II Systems (PacBio) using SMRTbell Express Template Prep Kit 2.0 kit (PacBio) under the manufacturer's instructions.

CCS reads from the raw sequencing results were generated from subreads by ccs (version 6.2.0) (https://github.com/nlhepler/pbccs) with the parameters ‘–skip-polish –min-passes 1 –min-rq 0.9′. To clean CCS reads, reads that contained barcode sequence and TSO sequence in CCS reads were selected for downstream analysis. The adapters at 5′ and 3′ ends were trimmed and the CCS reads were oriented from 5′ to 3′ direction. Multiple samples in one library will be extracted separately using Demultiplex (version 1.2.1) (https://github.com/jfjlaros/demultiplex) with parameters ‘-m 1 -d’. Duplicated CCS reads were removed according to the 12 random nucleotides at the 3′ end of CCS reads. Poly(A) tail length calculation was performed as previously reported (62). In brief, clean CCS reads were aligned to the mouse reference genome (mm9) using minimap2 (63) (version 2.15-r905) with parameters ‘-ax splice -uf -C5 –cs = short –secondary = no’. Gene exons were extracted from the refGene genome annotation file while overlapped exons were merged and duplicated exons were removed. CCS reads assigned to multiple genes were discarded. Polyadenylation analysis was performed using the TAPIS pipeline (64). CCS reads with trimmed poly(A) tail were aligned to the mouse reference genome (mm9) using GMAP (65) (version 2021-08-25). APA events were then analyzed using the run_tapis.py script in GMAP. For analysis of genes with altered distal APA, all sample reads were merged for APA calling. All reads aligned to annotated genes and carrying a poly(A) tail are used for analysis. To ignore minor heterogeneity in reads mapping at the poly(A) site, we computed the depth of each candidate poly(A) site based on the number of reads aligned within a five-nucleotide window. We adopted a greedy strategy, adding the site to the poly(A) sites list if it had a maximum read depth of at least two among the candidates and was outside the 15-nucleotide range of the previously added site. The site was subsequently excluded from the candidates’ list, and the algorithm continued until there were no remaining candidate sites. Reads with a poly(A) site within 10 bp around the nearest APA were counted as reads at this APA.

## Results

### FUS recognizes trimethylated H3K36

**Figure 1.**
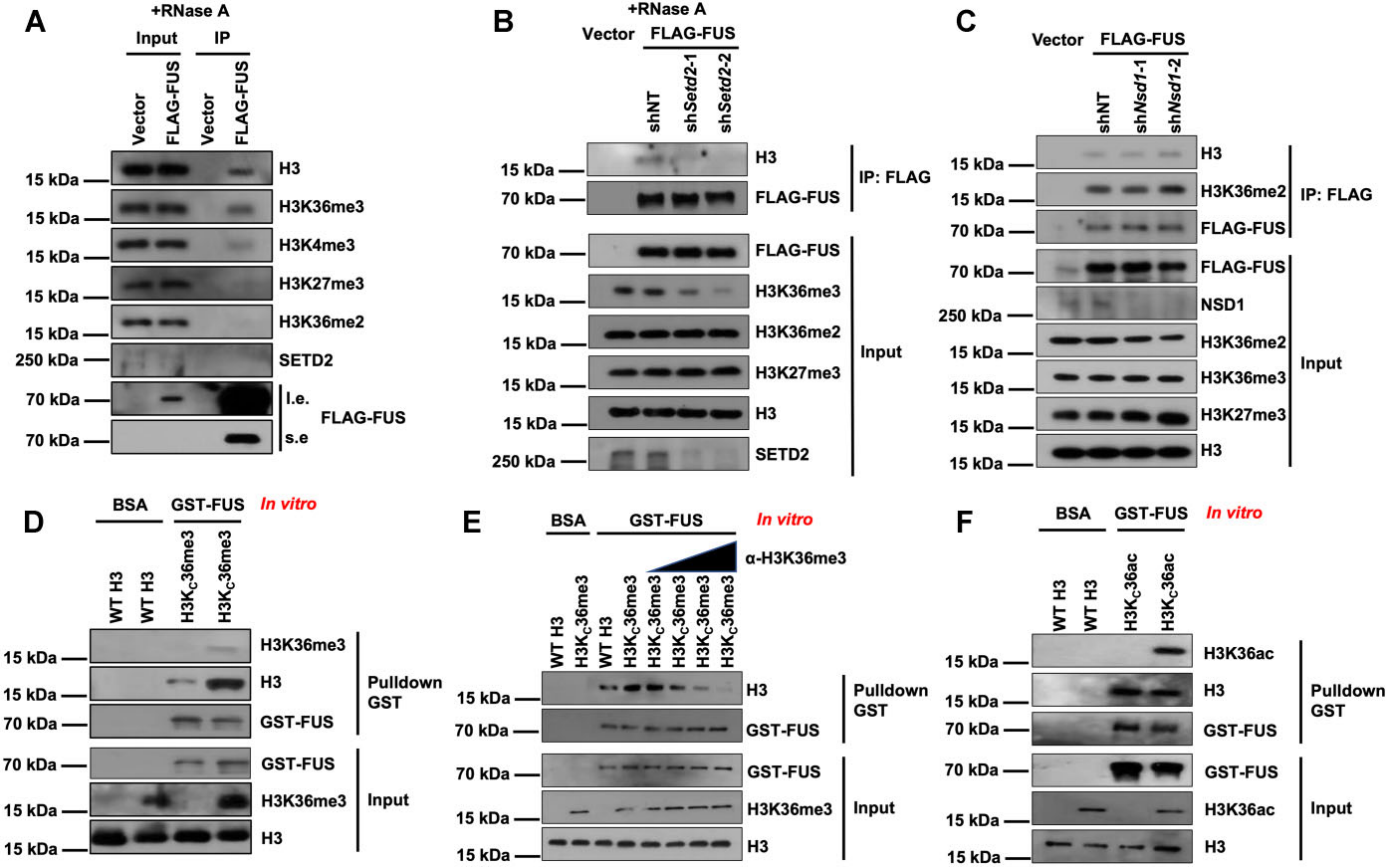
FUS recognizes histone H3K36me3. (**A**) Overexpressed FUS bound with H3. FUS was purified by FLAG IP in HEK293T cells overexpressing FLAG-tagged FUS. RNase A was used to digest RNA before IP. HEK293T cells transfected with empty vectors were used as negative controls. Proteins from input and IP samples were analyzed by Western blotting using the indicated antibodies. (**B**) Depletion of SETD2 decreased the binding between FUS and H3. RNase A was used to digest RNA before IP. *Setd2* was knocked down by two independent shRNA in HEK293T cells overexpressing FLAG-FUS. FLAG-FUS was purified by FLAG IP in cells. Proteins from input and IP samples were analyzed by Western blotting using the indicated antibodies. HEK293T cells that were infected with empty vectors were used as the negative control for IP experiment. NT, non-target control. (**C**) The depletion of NSD1 did not affect the binding between FUS and H3. *Nsd1* was knocked down by two independent shRNA. (**D**) FUS binds with more H3Kc36me3 nucleosomes than WT nucleosomes *in vitro*. WT and H3Kc36me3 nucleosomes were reconstituted *in vitro*. Recombinant GST-tagged FUS was purified and incubated with reconstituted nucleosomes. GST Seflnose Resin beads were used to pull down FUS. The input and beads-bound proteins were analyzed by Western blotting using the indicated antibodies. (**E**) H3K36me3 antibody repressed the binding between H3Kc36me3 nucleosome and FUS *in vitro*. Recombinant GST-tagged FUS was purified and incubated with an equal amount of WT or H3Kc36me3 histone as indicated. Different concentrations of antibodies were added to the incubated sample. GST Seflnose Resin beads were used to pull down FUS. The input and beads-bound proteins were analyzed by Western blotting using the indicated antibodies. (**F**) FUS has an equivalent binding affinity for both H3Kc36ac nucleosomes and WT nucleosomes *in vitro*. WT and H3Kc36ac nucleosomes were reconstituted *in vitro*. Recombinant GST-tagged FUS was purified and incubated with reconstituted nucleosomes. GST Seflnose Resin beads were used to pull down FUS. The input and beads-bound proteins were analyzed by western blotting using the indicated antibodies.

To investigate how H3K36me3 regulates polyadenylation, we aimed to test the proteins that recognize H3K36me3 and have mRNA-binding capacity. FUS was identified as a protein that fulfills these criteria based on previously published mass spectrometry results (66). We then overexpressed FUS in 293T cells and found that FUS bound to H3 and H3K36me3 was highly enriched in the analyzed histone marks (Figure 1A). SETD2 was not immunoprecipitated with the FUS protein, suggesting that the binding between FUS and histone H3 was not mediated by SETD2. Because FUS could bind with RNA, we performed the experiment with RNase A treatment. In addition, without RNase A treatment, FUS could bind to H3 (Supplemental Figure S1A). To further validate that FUS reads H3K36me3, we knocked down *Setd2*, which is critical for the trimethylation of H3K36, and performed immunoprecipitation (IP) with or without RNase A treatment. The results showed that the depletion of SETD2 abolished the binding between H3 and FUS (Figure 1B and Supplemental Figure S1B). When *No66* was knocked down or the cells were treated with KDM4A/B inhibitor (NSC636819) to increase H3K36me3, the binding between H3 and FUS was elevated (Supplemental Figure S1C and D). We knocked down *Nsd1*, which catalyzes the H3K36me2 modification in cells, and found that the interaction between H3 and FUS was not altered, indicating that the binding between FUS and H3 was not dependent on H3K36me2 (Figure 1C).

We designed an *in vitro* pulldown assay to analyze whether FUS recognizes H3K36me3. As reported previously (52), we reconstituted WT nucleosomes and H3Kc36me3 nucleosomes, which mimicked trimethylated H3K36 nucleosomes. Consistent with the *in vivo* IP results, trimethylation of H3K36 increased the interaction between the histone H3 and FUS (Figure 1D). The interaction between H3Kc36me3 and FUS was gradually blocked with an increased amount of anti-H3K36me3 antibodies (Figure 1E). In addition, the acetylation mimic at H3K36 showed no observed effect on the binding between FUS and H3 (Figure 1F). Together, these data suggest that FUS recognizes trimethylated H3K36.

### SETD2-catalyzed H3K36me3 recruits FUS to chromatin

**Figure 2.**
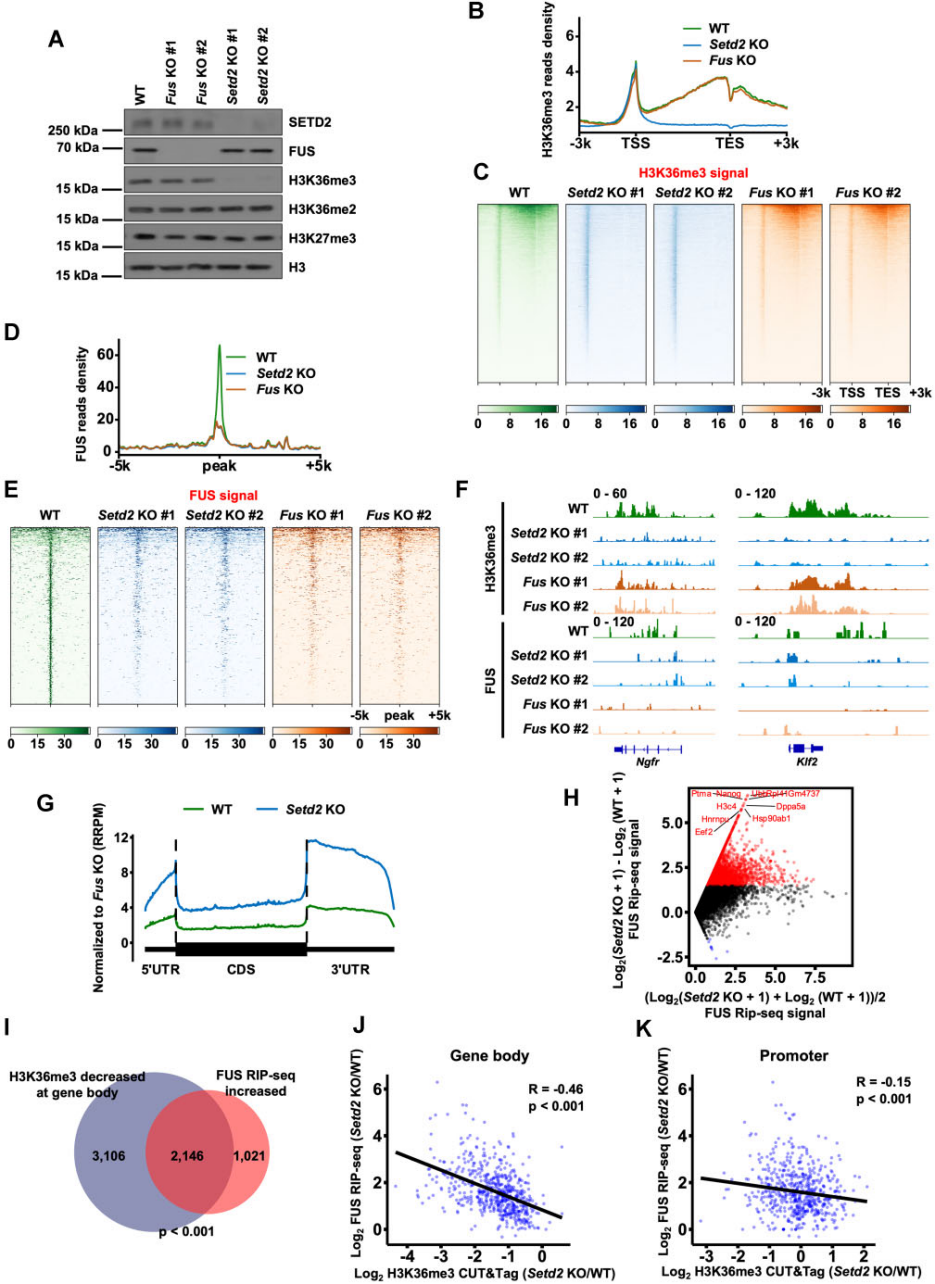
Depletion of H3K36me3 at gene bodies releases FUS from chromatin to RNA. (**A**) Western blotting result representing the total levels of indicated proteins in WT, *Fus* KO, and *Setd2* KO mESCs. Cell extracts were analyzed by western blotting using the indicated antibodies. (**B**) The normalized reads distribution profiles of H3K36me3 CUT&Tag spanning 3 kb of gene bodies in WT, *Fus* KO and *Setd2* KO mESCs. The average read density at all genes identified by NCBI RefSeq was plotted. TSS, transcription start site. TES, transcription end site. (**C**) Heatmaps showing H3K36me3 levels detected by H3K36me3 CUT&Tag around gene body regions in WT, *Fus* KO, and *Setd2* KO mESCs. 3 kb windows spanning the TSS to TES of all genes determined by NCBI RefSeq were plotted. Genes were organized by their enrichments of H3K36me3 in WT cells. (**D**) The normalized read distribution profiles of FUS CUT&Tag spanning 5 kb of WT FUS and H3K36me3 overlapped peaks in WT, *Fus* KO and *Setd2* KO mESCs. The average read density at all peaks was plotted. (**E**) Heatmaps showing FUS levels detected by FUS CUT&Tag around WT FUS and H3K36me3 overlapped peaks in WT, *Fus* KO and *Setd2* KO mESCs. 5 kb windows spanning the upstream and downstream of WT FUS and H3K36me3 overlapped peaks were plotted. Peaks were arranged by their enrichments of FUS in WT cells. (**F**) IGV tracks presenting the enrichment of H3K36me3 and FUS by CUT&Tag in WT, *Fus* KO, and *Setd2* KO mESCs. (**G**) The normalized read distribution profiles of FUS RIP-seq signals spanning gene bodies in WT and *Setd2* KO mESCs. The average read density at all genes identified by NCBI RefSeq was plotted. RIP-seq signals were normalized by the spiked-in HeLa cells first and then normalized by the signals in *Fus* KO cells. UTR, untranslated regions. CDS, coding sequence. RRPM, reference-adjusted reads per million. (**H**) The scatter plot showing the changes of FUS RIP-seq signals between WT and *Setd2* KO mESCs. RIP-seq signals were normalized by the spiked-in HeLa cells first and then normalized by the signals in *Fus* KO cells. The top 10 genes with increased FUS RIP-seq signals were shown. (**I**) Venn diagram illustrating the overlap of H3K36me3 signal decreased genes and FUS RIP-seq signal increased genes in *Setd2* KO mESCs when compared with WT mESCs. *P* value was determined by Fisher's exact statistical test, two-sided. (**J**) Correlations between the altered signals of H3K36me3 CUT&Tag and FUS RIP-seq at gene bodies in *Setd2* KO mESCs compared with WT mESCs. R, correlation coefficients that were assessed by Pearson product-moment correlation. *P* values were calculated by paired *t*-test, two-sided. (**K**) Same as in (J), except signals at promoters were calculated.

We then sought to determine how FUS reads H3K36me3 genome-wide. In addition to two previously created independent *Setd2*-knockout (KO) mESC lines (43), we generated two *Fus*-KO mESC lines using two independent sgRNAs bearing 2 and 4 base-pair deletions (Supplemental Figure S2A). While *Setd2* KO decreased the total level of H3K36me3, *Fus* KO did not lead to a detectable change in the total level of H3K36me3, as determined by Western blotting (Figure 2A). In addition, *Fus* KO did not affect the activity of alkaline phosphatase in mESCs but led to slower cell proliferation of mESCs (Supplemental Figure S2B and C). As reported before (43), the expressions of pluripotent marks, *Oct4* and *Nanog*, were unchanged in *Setd2* KO mESCs (Supplemental Figure S2D). To analyze the enrichment of H3K36me3 and FUS on chromatin, we performed H3K36me3 and FUS CUT&Tag with WT, *Fus* KO, and *Setd2* KO mESCs. Two independent replicates of each KO cell line were highly correlated, and the data obtained with each line were merged for further analysis (Supplemental Table S1). We analyzed the enrichment of H3K36me3 in genomic regions spanning 3 kb upstream and downstream of the gene body in all genes that had been identified via NCBI RefSeq data. The sequencing results from two KO cell lines were merged to increase the strength of the analyses (Figure 2B). As previously reported (43), *Setd2* KO abolished the enrichment of H3K36me3 at gene bodies but not at promoter regions. *Fus* KO did not affect the overall enrichment of H3K36me3 throughout gene regions. This observation was confirmed by a heatmap showing H3K36me3 signals spanning each gene in individual cell lines (Figure 2C). In addition, FUS was enriched at the promoter and gene body regions like H3K36me3 (Supplemental Figure S2E and F). FUS signals were all abolished at the promoter and gene body regions in *Fus* KO mESCs, demonstrating the specificity of FUS CUT&Tag. Depletion of SETD2 abolished the enrichment of FUS at gene body regions but not at promoter regions. Because it has been reported that RNA Polymerase II (Pol II) can recruit FUS to the chromatin (67), it is possible that the retention of FUS at promoters is regulated by Pol II. To further analyze how FUS reads SETD2-catalyzed H3K36me3, we first called FUS and H3K36me3 overlapped peaks in WT cells and then calculated the enrichment of FUS in different cell lines (Figure 2D and E). When *Setd2* was knocked out, the enrichment of FUS at H3K36me3 peaks was decreased to levels similar to those in *Fus* KO cells, representing the background signals of FUS CUT&Tag. Visualization with IGV (Integrative Genomics Viewer) also revealed that FUS was abolished at H3K36me3-enriched regions when *Setd2* was knocked out (Figure 2F). Moreover, two independent KO cell lines showed a similar signal distribution pattern, further demonstrating the reproducibility of the sequencing results.

Because it has been proposed that H3K36me3 promotes PRC2 complex recruitment to increase H3K27me3 (68), we profiled H3K27me3 in WT, *Setd2* KO and *Fus* KO cells. The enrichment of H3K27me3 at promoter regions was not altered when *Setd2* or *Fus* was knocked out (Supplemental Figure S2G and H). The IGV visualization also revealed that the H3K27me3 signals were not changed in *Setd2* KO or *Fus* KO cells compared with those in WT cells (Supplemental Figure S2I).

RNA-binding ability is suggested to be crucial for FUS function (69). We next asked how *Setd2* KO affected FUS binding with RNA. We performed FUS RIP-seq (RNA immunoprecipitation sequencing) to profile FUS binding RNA in WT, *Setd2* KO and *Fus* KO cells. To normalize FUS signals among different cell lines, we spiked HeLa cells as internal controls. The RIP-seq signals in *Fus* KO cells were used as background controls. Signals that were first normalized to spike-in RNA and then compared to those in *Fus* KO cells were considered true signals. Two independent replicates were established, and the data from these replicates were merged for further analysis (Supplemental Table S1). The total levels of gene expression were not affected by the *Setd2* KO or *Fus* KO (Supplemental Figure S2J). In line with the decrease in chromatic enrichment, the average RNA-binding signal of FUS at all genes was increased in *Setd2* KO cells (Figure 2G). Similarly, FUS-binding signals at each gene were mostly increased in *Setd2* KO cells (Figure 2H). Moreover, genes with decreased H3K36me3 abundance at gene bodies largely overlapped with genes showing increased FUS signals (Figure 2I). To analyze whether the increase in FUS binding with RNA was correlated with the loss of H3K36me3 genome-wide, we calculated genome-wide correlations between changes in H3K36me3 enrichment and FUS binding at each gene in WT and *Setd2* KO cells. H3K36me3 alterations at gene body regions (*R* = –0.46), but not those at promoters (*R* = –0.15), were highly correlated with changes in FUS binding with RNA, indicating that the increase in FUS and RNA interaction was directly caused by SETD2 depletion (Figure 2J and K).

We had previously found that SMYD5 was critical for the enrichment of H3K36me3 at promoter regions, and this SMYD5 dependence may affect the binding between FUS and RNA (43). We, therefore, performed FUS RIP-seq with WT and *Smyd5* KO mESCs to analyze whether *Smyd5* KO affects FUS binding with RNA. Two independent replicates were highly correlated, and the data of these replicates were merged for further analysis (Supplemental Table S1). Consistent with the observation that H3K36me3 alterations at promoters were not correlated with changes in the FUS–RNA binding signal in *Setd2* KO mESCs, *Smyd5* KO did not affect overall FUS binding with RNA (Supplemental Figure S2K). Additionally, depletion of SETD2 resulted in the relocation of FUS from the nucleus to the cytoplasm in approximately 30% of cells (Supplemental Figure S2L-N). Together, these data suggest that SETD2-catalyzed H3K36me3 recruits FUS to chromatin and, after SETD2 is abolished, FUS is released from chromatin and binds with RNA.

### Dysregulation of FUS leads to an increase in APA far from stop codons

**Figure 3.**
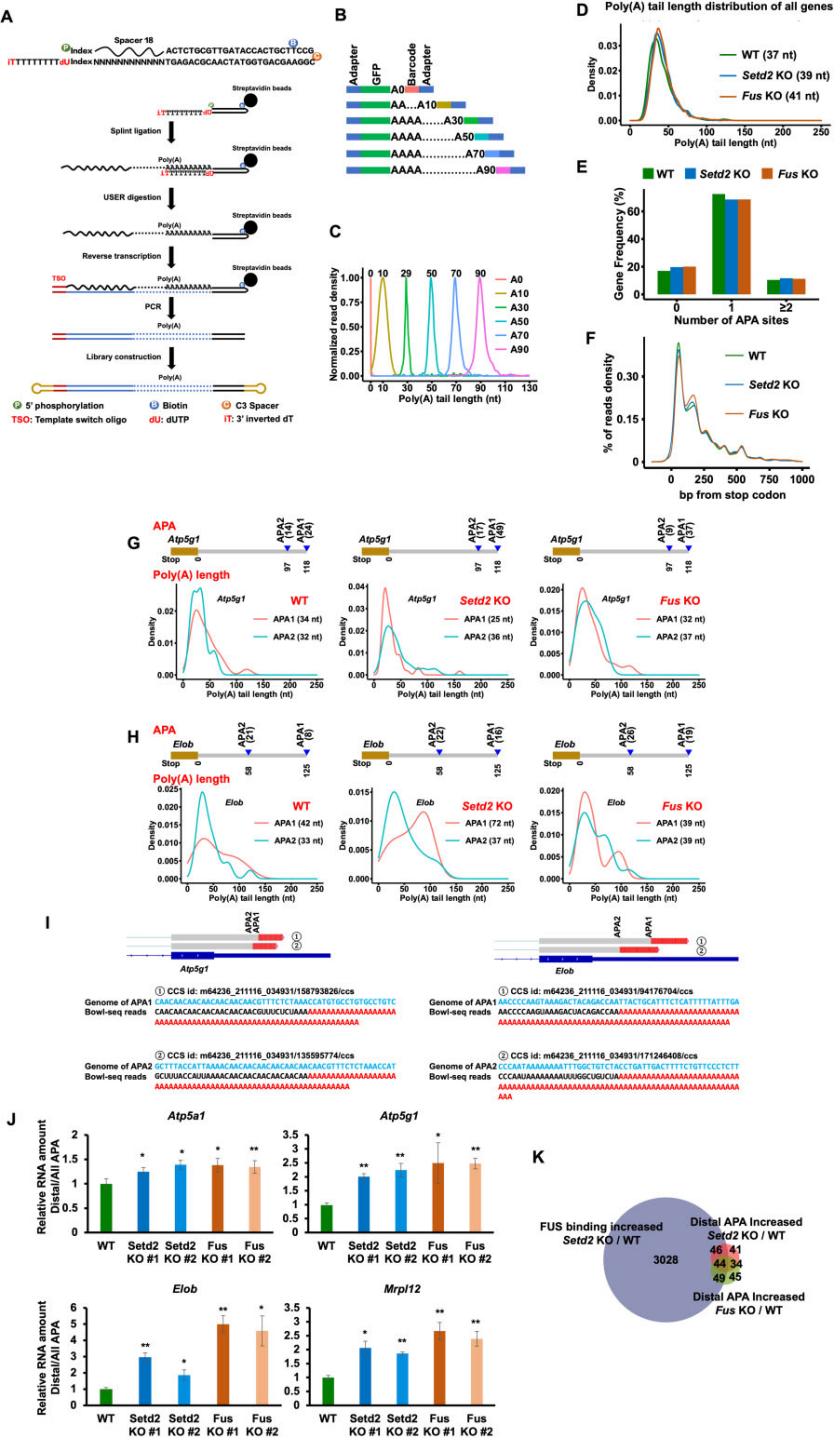
Dysregulation of FUS leads to increased distal APA. (**A**) Schema illustrating the process of Bowl-seq. See the method for a detailed description of the process. (**B**) Schema showing the designed poly(A) tail spike-in. GFP sequence with a defined length of poly(A) was followed by a barcode to be split for analysis. (**C**) The median poly(A) tail length of each spike-in measured by Bowl-seq. (**D**) Global distribution of poly(A) tail lengths of all genes. The median poly(A) tail length of the genes for each cell line was shown. (**E**) APA events of transcripts detected by Bowl-seq. APA site = 0 presented genes that were not detected with a defined poly(A) site. (**F**) Distribution of the reads density with APA at the stop codon region. (**G**) The reads distributions at two APA in the *Atp5g1* gene. The number of APA reads was shown at the right of the APA model. Proximal and Distal APA reads were indicated in the APA sites and the APA was calculated by the reads number. The median length of poly(A) tails from APA was shown in the graph. (**H**) Same as in (G), except the *Elob* gene was shown. (**I**) Illustration showing the mapped Bowl-seq reads with 3′ end of genes. Two examples of bowl-seq reads mapping to the 3′end of the annotated genes were shown. APA, alternative polyadenylation. Blue letters represent genomic sequences, red letters represent poly(A), and black letters represent mRNA sequences. (**J**) RT-qPCR results showing the different polyadenylation levels of genes in WT, *Setd2* KO and *Fus* KO mESCs. The ratio of distal APA in the total APA RNA in WT cells was normalized as 1. The data were represented by the mean ± SD (*N* = 3 independent replicates). * *P* < 0.05 and ** *P* < 0.01, as determined by paired *t*-test, one-sided. (**K**) Venn diagram showing the overlap of genes with increased distal APA in *Setd2* KO and *Fus* KO mESCs, and genes with elevated FUS binding in *Setd2* KO mESCs.

Since H3K36me3 and FUS were enriched at the 3′ end of genes, we designed to detect the poly(A) in cells. High-quality sequencing of repeated nucleotides is necessary to characterize the poly(A). However, the second generation of high-throughput sequencing is not suitable for this requirement. We then considered utilizing the long-read sequencing platform SMRT (single molecule, real-time) sequencing to profile mRNA poly(A). SMRT sequencing overcomes shortages due to the low read quality of repetitive sequences in second-generation sequencing. To detect the exact length of the poly(A) tail in mRNA, we designed a self-annealed oligo to facilitate splint enzyme ligation, which is more efficient than other ligation enzymes. A dUTP was synthesized after a polydT and can be digested by a USER (uracil-specific excision reagent) enzyme, releasing the polydT in the oligo before reverse transcription. In addition, an inverted dT was synthesized at the 3′ end to prevent elongation of undigested oligos. The reverse-transcribed cDNA was PCR amplified and ligated with adaptors in preparation for SMRT sequencing (Figure 3A; see the details of library construction steps in the Methods). We called this method biotinylated oligo-coupled polyadenylation profiling with long-read sequencing (Bowl-seq).

We then applied Bowl-seq to identify poly(A) changes in WT, *Setd2* KO, and *Fus* KO mESCs. To test the accuracy of the poly(A) read length, we spiked synthesized GFP DNA with a defined poly(A) length in the sequencing library (Figure 3B). The recovered GFP reads in the library were separated on the basis of their identical indices and subjected to poly(A) length calculation. The median lengths of the measured poly(A) sequences were the same as those spiked in the DNA, demonstrating the accuracy of the sequencing results (Figure 3C). The detected numbers of unique circular consensus sequencing (CCS) reads were similar among different cell lines (Supplemental Figure S3A). Two independent replicates of each tested cell line were highly correlated with gene poly(A) lengths, and the data were merged for further analysis (Supplemental Figure S3B). The data from two independent KO cell lines were also merged to increase the reproducibility of the data analysis. The poly(A) lengths in the CCS reads were not changed in *Setd2*-KO and *Fus*-KO mESCs compared with those in WT mESCs (Supplemental Figure S3C). We calculated the poly(A) length of each gene and found that the distributions and median value of the poly(A) length were not changed in *Setd2* KO or *Fus* KO mESCs (Figure 3D). We noticed the poly(A) tail length is not so long as to 250 adenosines in oocyte cells. In line with this, previous TAIL-seq results showed that the median lengths were 60 nt and 59 nt in NIH 3T3 and HeLa, respectively (8). The median length of poly(A) may vary among different cell lines. Moreover, the poly(A) length of the individual genes was not significantly changed in the tested cell lines (Supplemental Figure S3D). These data suggest that loss of H3K36me3 or FUS did not affect the poly(A) length in mESCs. 

We then analyzed whether the APA of mRNA was affected upon loss of *Setd2* or *Fus*. APA generates diverse 3′ UTRs, resulting in altered stability of transcripts. The frequency of genes with different numbers of APA was similar in the WT, *Setd2* KO and *Fus* KO mESCs (Figure 3E). Interestingly, we found that the APA was mainly located at sites distant from stop codons in *Setd2* and *Fus* KO mESCs, in contrast to their location in WT mESCs (Figure 3F). To compare the statistics among the distributions of APA reads, we used a 50 bp to a stop codon to conduct a Fisher's extract *P* value test. The *P* value between WT and *Setd2* KO mESCs was 0.025 and 4.49 × 10^-13^ between WT and *Fus* KO mESCs, showing significantly increased distal APA selection in the *Setd2* KO and *Fus* KO mESCs. Please note the proximal and distal APA reads in each gene were defined as the approach described in the methods. In addition, the usage of non-A residues in the poly(A) tail was increased in the *Setd2* KO and *Fus* KO mESCs (Supplemental Figure S3E and F). The RNA splicing events remained unchanged in mESCs with *Setd2* KO (70) and *Fus* KO (Supplemental Figure S3G).

We then investigated the biological functions of genes with increased distal APA with a Gene Ontology (GO) analysis. Several hits indicated that the affected genes in both *Setd2* and *Fus* KO mESCs were enriched in RNA processing and regulation of mitochondrial function (Supplemental Figure S3H and I). Two genes with increased distal APA selection sites were shown as examples. In *Setd2* and *Fus* KO mESCs, *Atp5g1*, which encodes a membrane subunit of mitochondrial ATP synthase, showed an elevated number of reads with distal APA (Figure 3G). In another example, we found that *Elob*, a subunit of the transcription Factor B complex that increases Pol II transcription elongation past arrest sites, exhibited an increased distribution of reads with distal APA (Figure 3H). Representative Bowl-seq reads were shown to identify different APA (Figure 3I). In addition, the increase of distal APA was further confirmed in *Setd2* and *Fus* KO cell lines by using RT-qPCR (Figure 3J). Genes with increased distal APA largely overlapped between *Setd2* KO and *Fus* KO mESCs (Figure 3K). Moreover, in *Setd2* KO and *Fus* KO mESCs, the genes showing increased distal APA significantly overlapped with genes with increased FUS binding in *Setd2* KO cells as determined by RIP-seq, suggesting that the increase in reads in distal APA was caused by elevated FUS binding.

We further analyzed the expression of several genes with increased distal APA that were associated with mitochondrial functions and mRNA processing. The expression levels of these genes, including *Atp5a1*, *Atp5g1*, *Elob* and *Mrpl12*, were elevated in two independent *Setd2-* and *Fus* KO cell lines, as detected by RT−qPCR (Supplemental Figure S3J). In addition, genes that have an increased distal APA and FUS binding showed elevated expressions genome-wide in *Setd2* KO and *Fus* KO mESCs (Supplemental Figure S3K). The way FUS impacts APA selection depends on the position of their binding site in relation to the poly(A) site (27). We conducted an analysis of the enrichments of FUS and H3K36me3 at both proximal and distal poly(A) sites. In the case of genes with altered APA, FUS had a higher enrichment at proximal poly(A) sites compared to distal ones. Furthermore, H3K36me3 also showed higher enrichment at proximal poly(A) sites (Supplemental Figure S3L). This finding was consistent with previous research indicating that FUS, located upstream of an alternative polyadenylation site, downregulated the expression of short transcripts and inhibited polyadenylation (23). Together, these results demonstrate that the dysregulation of FUS binding, either by depletion of SETD2 or FUS, led to an increased number of APA distal from gene stop codons.

### Proline residues in the ZNF domain are critical for the recognition of trimethylated H3K36

**Figure 4.**
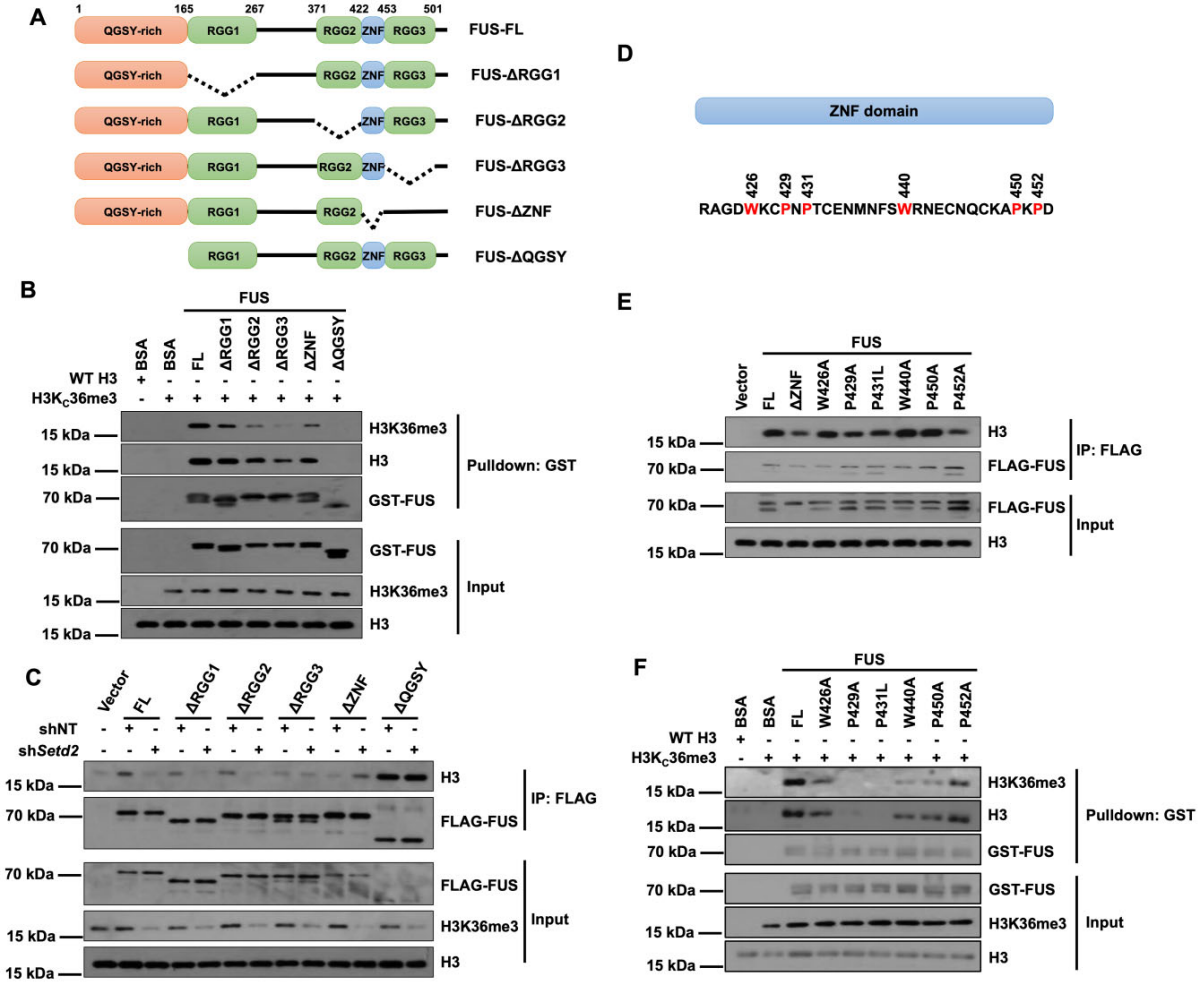
Prolines in the ZNF domain regulate FUS and H3K36me3 interaction. (**A**) An illustration showing the protein domains of FUS. (**B**) Deletion of functional domains of FUS reduced its interaction with H3Kc36me3 octamers *in vitro*. Recombinant GST-tagged FUS and FUS mutants were purified and incubated with an equal amount of WT or H3Kc36me3 histone core octamers as indicated. Glutathione agarose beads were used to pull down FUS. The input and beads-bound proteins were analyzed by Western blotting using the indicated antibodies. (**C**) RGG3 and ZNF domains were important for the binding between FUS and H3 *in vivo*. *Setd2* was knocked down by shRNA in HEK293T cells. FLAG-tagged WT and mutant FUS were overexpressed in control and SETD2 depleted cells and purified by FLAG IP. Proteins from input and IP samples were analyzed by Western blotting using the indicated antibodies. NT, non-target control. (**D**) An illustration showing the amino acid residues of the ZNF domain of FUS. Proline (P) and tryptophan (W) residues were labeled with red. (**E**) Prolines in the ZNF domain were important for the binding between FUS and H3 *in vivo*. FLAG-tagged WT and mutant FUS were overexpressed in HEK293T cells and purified by FLAG IP. Proteins from input and IP samples were analyzed by Western blotting using the indicated antibodies. HEK293T cells which were infected with empty vectors were used as the negative control for IP experiment.(**F**) Proline and tryptophan in the ZNF domain decreased the interaction between FUS and H3Kc36me3 octamers *in vitro*. Recombinant GST-tagged FUS and FUS mutants were purified and incubated with an equal amount of WT or H3Kc36me3 histone core octamers as indicated. Glutathione agarose beads were used to pull down FUS. The input and beads-bound proteins were analyzed by Western blotting using the indicated antibodies.

We further investigated how FUS recognized H3K36me3. FUS harbors several domains, including a QGSY-rich domain, RGG1-3 domains, and a ZNF domain (Figure 4A). We purified full-length FUS (FUS-FL), a QGSY domain deletion FUS mutant (FUS-ΔQGSY), RGG1-3 domain deletion FUS mutants (FUS-ΔRGG1, FUS-ΔRGG2 and FUS-ΔRGG3), and a ZNF domain deletion FUS mutant (FUS-ΔZNF) and tested the importance of these domains in the interactions between FUS and H3 *in vitro*. Since the ZNF domain carries a potential DNA-binding domain, we reconstituted WT and H3Kc36me3 octamers, in which the ‘601’ DNA needed for nucleosome reconstitution was excluded, to perform pulldown assays. The results showed that depletion of any of these domains reduced the binding between FUS and the H3Kc36me3 octamers (Figure 4B). To further demonstrate how different domains in FUS recognize H3K36me3 *in vivo*, we overexpressed mutant FUS with a FLAG tag in HEK293T cells with or without *Setd2* knocked down, purified FLAG-tagged FUS, and then analyzed the copurified proteins by Western blotting (Figure 4C). FUS-ΔQGSY showed increased interaction with H3, whereas the other mutant FUS proteins exhibited reduced binding with H3. In addition, SETD2 depletion did not reduce H3 binding with FUS-ΔRGG3, FUS-ΔZNF, or FUS-ΔQGSY. Depletion of SETD2, however, led to increased binding between FUS-ΔZNF and H3, possibly through a compensatory effect. We also analyzed the cellular localization of the mutant FUS proteins. FUS-ΔZNF was distributed to the cytoplasm, whereas the other mutant FUS proteins were localized mainly in the nucleus (Supplemental Figure S4A). The QGSY region is a domain that contains low-complexity sequences and plays a crucial role in the self-assembly process of FUS. The RGG1/2 domains can impact the RNA-binding ability of FUS. Mutations in these domains, *in vivo*, may influence the protein's behavior, which could ultimately affect the binding between H3 and FUS. The *in vitro* experiments used purified proteins and octamers without DNA and RNA to perform pull-down, which simplified the interactions between FUS and H3. Together, these data indicate that the RGG3 and ZNF domains are important for FUS recognition of H3K36me3.

RGG domains are methylated to regulate the phase separation of FUS, which may indirectly affect the binding between FUS and H3K36me3. We thus focused on the ZNF domain, which has not been reported to directly bind H3K36me3. Several domains have been proposed to bind trimethylated H3K36, including the PWWP domain, PHD finger domain, Chromo domain, and Tudor domain. We noticed several proline (P) and tryptophan (W) residues in the ZNF domain, corresponding to the P and W signatures of the PWWP domain (Figure 4D). In addition, an ALS patient bearing a P431L mutation has been reported (71). It is possible that these P and W residues facilitate the interaction between FUS and H3K36me3. We mutated every P and W amino acid in the ZNF domain and overexpressed the mutant proteins to analyze their interactions with H3. P431 was replaced with an L residue to mimic the mutation in the ALS patient. Similar to ZNF domain deletion, the FUS-P429A and FUS-P431L mutations decreased the binding between FUS and H3 *in vivo*, while FUS-P452A mutation showed a moderate effect (Figure 4E). In addition, the FUS-P429A, FUS-P431L and FUS-P452A mutant proteins were localized in the cytoplasm as foci (Supplemental Figure S4B and C). To confirm that the reduced binding of the FUS mutants with H3 was not caused by decreased DNA binding, we purified mutant FUS proteins and analyzed their binding with H3Kc36me3 octamers (Figure 4F). All the tested mutants decreased the binding between FUS and H3Kc36me3 octamers. More importantly, FUS-P429A and FUS-P431L showed the lowest binding affinity *in vitro*. We focused on the consistently changed results in these *in vivo* and *in vitro* experiments and identified FUS-P429A and FUS-P431L showed a more reliable impact. Because the ZNF domain may affect the RNA binding ability of FUS, we also designed to test the RNA binding affinities of these mutant proteins. We used microscale thermophoresis (MST) to analyze the association of FUS ZNF deletion and point mutations with two primary sequence motifs recognized by FUS (GGUG and UUAGGG) (25,72,73). The results showed that these mutations had a minimal impact on RNA affinity, and in fact, P421L even showed a slight increase in affinity with the GGUG sequence (Supplemental Figure S4D). Overall, we did not observe a significant decrease in RNA affinity with the ZNF domain deletion or point mutations based on the two tested sequences. Altogether, these data indicate that the ZNF domain in FUS is critical for the recognition of trimethylated H3K36, and the proline residues at positions 429 and 431 are critical.

### Mutations of proline residues in the ZNF domain lead to an increase in APA distant from stop codons

**Figure 5.**
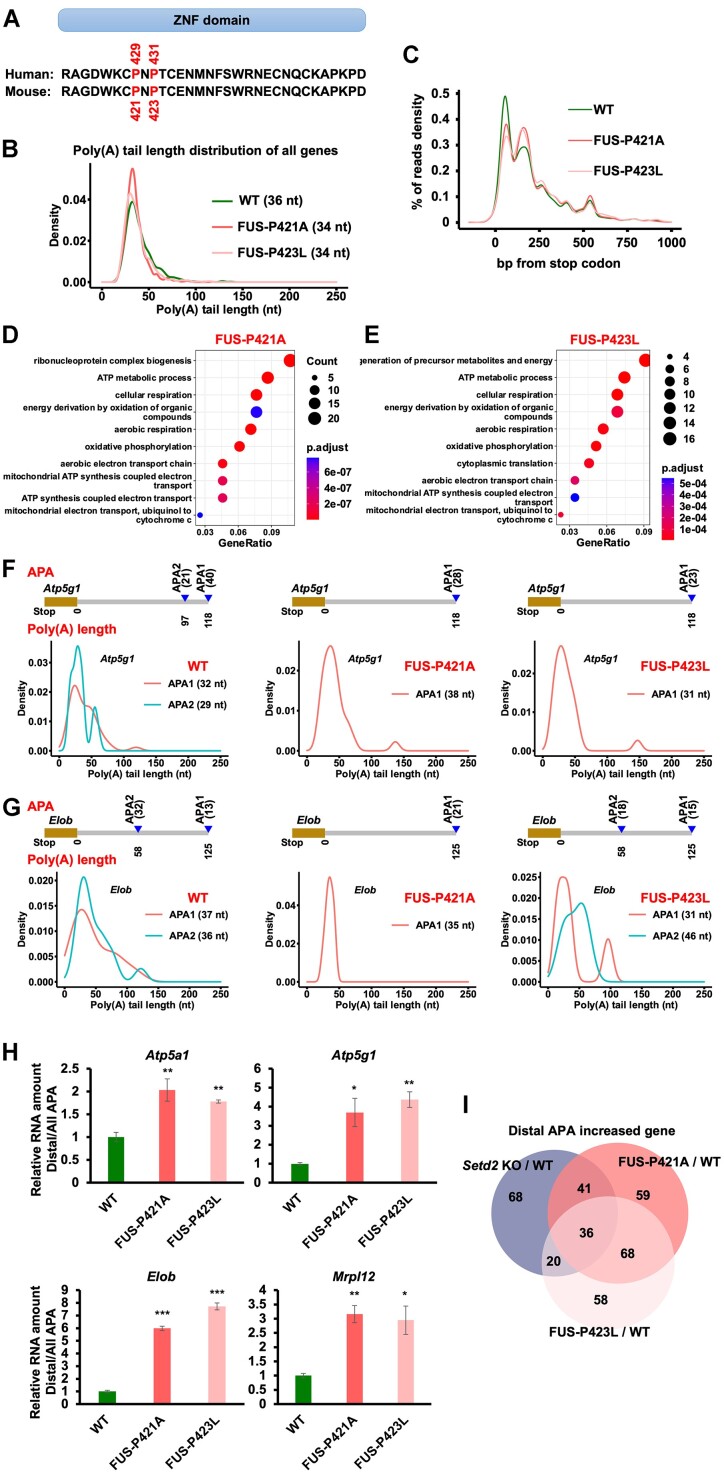
Loss of H3K36me3 recognition of FUS leads to increased distal APA. (**A**) Schema illustrating the position of amino acid residues in human and mouse FUS protein. (**B**) Global distribution of poly(A) tail lengths of all genes. The median poly(A) tail length of the genes for each cell line was shown. (**C**) Distribution of the reads density with APA at the stop codon region. (D and E) GO terms showing the genes with increased distal APA selection in FUS-P421A mESCs (**D**) and FUS-P423L mESCs (**E**). (**F**) The reads distributions at two APA in the *Atp5g1* gene. The number of APA reads was shown at the right of the APA model. Proximal and Distal APA reads were indicated in the APA sites and the APA was calculated by the reads number. The median length of poly(A) tails from APA was shown in the graph. (**G**) Same as in (G), except the *Elob* gene was shown. (**H**) RT-qPCR results showing the different polyadenylation levels of genes in WT and FUS mutant mESCs. The ratio of distal APA in the total APA RNA in WT cells was normalized as 1. The data were represented by the mean ± SD (*N* = 3 independent replicates). * *P* < 0.05, ** *P* < 0.01 and *** *P* < 0.01 as determined by paired *t*-test, one-sided. (**I**) Venn diagram showing the overlap of genes with increased distal APA in *Setd2* KO and FUS mutant mESCs.

Since the FUS-P429A and FUS-P431L mutant proteins showed the lowest binding with H3Kc36me3 *in vitro*, we aimed to knock in these mutations and measure the effect in mESCs. The ZNF domain of FUS is conserved between humans and mice. The P429 and P431 residues in the human FUS protein correspond to the P421 and P423 residues in the mouse FUS protein, respectively (Figure 5A). We utilized the CRISPR/Cas9 system and knocked in FUS-P421A and FUS-P423L in mESCs (Supplemental Figure S5A and B). The FUS mutations didn’t affect the activity of alkaline phosphatase but led to a lower cell proliferation rate of mESCs (Supplemental Figure S5C and D). In addition, the FUS-P421A and FUS-P423L mutant proteins were more enriched in the cytoplasm than WT FUS (Supplemental Figure S5E). The total levels of gene expression were not affected by the FUS-P423L mutation (Supplemental Figure S5F).

We conducted Bowl-seq to analyze how poly(A) length and APA were affected by FUS mutations. Similar numbers of CCS reads and recovered genes were detected among WT, FUS-P421A and FUS-P423L mESCs (Supplemental Figure S6A). Two independent replicates of each tested cell line were highly correlated, and the data were merged for further analysis (Supplemental Figure S6B). The overall poly(A) lengths of CCS reads and genes were not changed in the tested cell lines (Figure 5B and Supplemental Figure S6C). In addition, the poly(A) length of each gene was not significantly changed (Supplemental Figure S6D). These data were consistent with the results obtained with the *Setd2* and *Fus* KO cells: the RNA poly(A) length was not affected.

We then analyzed how APA was affected in FUS mutant mESCs. The percentage of genes with different numbers of APA was not changed in the FUS mutant mESCs (Supplemental Figure S6E). More importantly, we detected elevated enrichment of reads with an APA distal to a stop codon in the FUS-P421A and FUS-P423L mutant mESCs (Figure 5C). To compare the statistics among the distributions of APA reads, we used a 50 bp to a stop codon to conduct a Fisher's extract p value test. The APA were significantly enriched in the distal region from a stop codon in FUS mutant mESC (Fisher's extract p value was 4.2 × 10^-20^ between WT and FUS-P421A mESCs and was 4.59 × 10^-13^ between WT and FUS-P423L mESCs). In addition, the usage of non-A residues in the poly(A) tail was increased in FUS mutant mESCs (Supplemental Figure S6F and G). The RNA splicing events remained unchanged in FUS-P423L mESCs (Supplemental Figure S6H). The distal APA increased and FUS-P421L binding increased genes were elevated in FUS-P423L mESCs (Supplemental Figure S6I). The GO analysis showed that genes with increased distal APA were highly enriched in the regulation of mitochondrial function (Figure 5D and 5E). Two genes, *Atp5g1* and *Elob*, were shown with their APA highlighted. More reads were distributed at distal APA in these two genes (Figure 5F and G). The increase of distal APA in four genes was further confirmed by RT-qPCR (Figure 5H). Most importantly, the genes with increased reads distributed at distal APA largely overlapped among *Setd2* KO, FUS-P421A mutant, and FUS-P423L mutant mESCs (Figure 5I). Together, these data suggest that FUS-P421A and FUS-P423L mutations led to increased APA distal to a stop codon, which was similar to the site selection after depletion of SETD2 in mESCs.

### P423L mutation abolishes FUS binding with H3K36me3 on chromatin and increases FUS binding with RNA

**Figure 6.**
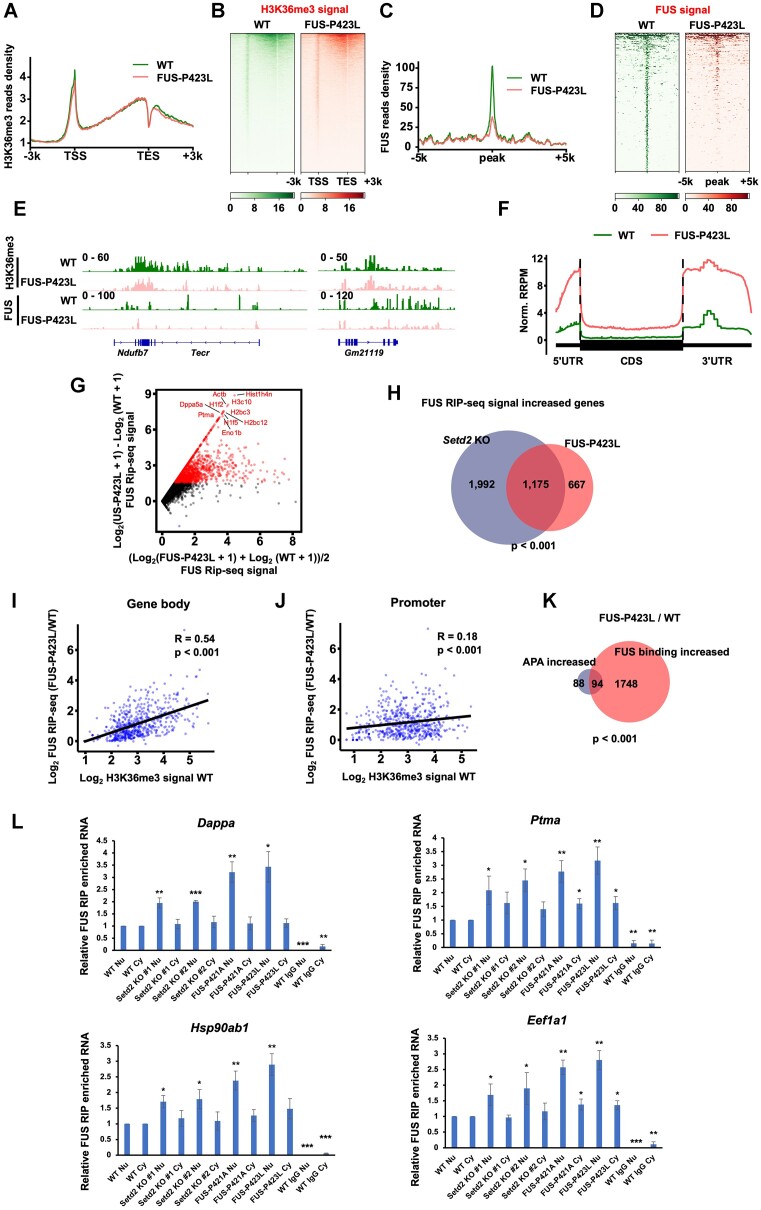
FUS-P423L mutation represses FUS binding with H3K36me3 to increase its RNA binding. (**A**) The normalized reads distribution profiles of H3K36me3 CUT&Tag spanning 3 kb of gene bodies in WT and FUS-P423L mutant mESCs. The average read density at all genes identified by NCBI RefSeq was plotted. TSS, transcription start site. TES, transcription end site. (**B**) Heatmaps showing H3K36me3 levels detected by H3K36me3 CUT&Tag around gene body regions in WT and FUS-P423L mutant mESCs. 3 kb windows spanning the TSS to TES of all genes determined by NCBI RefSeq were plotted. Genes were organized by their enrichments of H3K36me3 in WT cells. (**C**) The normalized read distribution profiles of FUS CUT&Tag spanning 5 kb of WT FUS and H3K36me3 overlapped peaks in WT and FUS-P423L mutant mESCs. The average read density at all peaks was plotted. (**D**) Heatmaps showing FUS levels detected by FUS CUT&Tag around WT FUS and H3K36me3 overlapped peaks in WT and FUS-P423L mutant mESCs. 5 kb windows spanning the upstream and downstream of WT FUS and H3K36me3 overlapped peaks were plotted. Peaks were arranged by their enrichments of FUS in WT cells. (**E**) IGV tracks presenting the enrichment of H3K36me3 and FUS by CUT&Tag in WT and FUS-P423L mutant mESCs. (**F**) The normalized read distribution profiles of FUS RIP-seq signals spanning gene bodies in WT and FUS-P423L mutant mESCs. The average read density at all genes identified by NCBI RefSeq was plotted. RIP-seq signals were normalized by the spiked-in HeLa cells. UTR, untranslated regions. CDS, coding sequence. Norm. RRPM, normalized reference-adjusted reads per million. (**G**) The scatter plot showing the changes of FUS RIP-seq signals between WT and FUS-P423L mutant mESCs. RIP-seq signals were normalized by the spiked-in HeLa cells. Top 10 genes with increased FUS RIP-seq signals were shown. (**H**) Venn diagram illustrating the overlap of genes with increased FUS RIP-seq signals in FUS-P423L and *Setd2* KO cells compared with WT cells. *P* value was determined by Fisher's exact statistical test, two-sided. (**I**) Correlations between the H3K36me3 CUT&Tag signal in WT mESCs and altered signals of FUS RIP-seq at gene bodies in FUS-P423L mutant mESCs compared with WT mESCs. R, correlation coefficients that were assessed by Pearson product-moment correlation. *P* values were calculated by paired t-test, two-sided. (**J**) Same as in (I), except signals at promoters were calculated. (**K**) Venn diagram presenting the overlap between genes with altered distal APA and elevated FUS binding. (**L**) RT-qPCR analysis of FUS-bound RNA levels in the nucleus and the cytoplasm upon *Setd2* KO and FUS mutation. Four genes that were the top genes with increased FUS RIP-seq signals were analyzed. Nu, nucleus. Cy, cytoplasm. * *P* < 0.05, ** *P* < 0.01 and *** *P* < 0.01 as determined by paired t-test, one-sided. RIP assays were conducted in the cell nucleus and cytoplasm separately. IgG was used as the negative control.

Since the FUS-P423L mutation has been found in an ALS patient, we analyzed the enrichment of H3K36me3 and FUS protein in WT and FUS-P423L mutant mESCs. Two independent replicates were established, and they were found to be highly correlated (Supplemental Table S1). We then merged the data of the two replicates for further analysis. The FUS-P423L mutation did not alter the distribution of H3K36me3 in gene body regions (Figure 6A and B). When FUS was mutated to FUS-P423L, the enrichment of FUS was largely abolished at gene bodies but not at promoters (Supplemental Figure S7A and S7B), which was consistent with the observation that *Setd2* KO reduced the enrichment of FUS at gene bodies but not promoters (Supplemental Figure S2E and F). To further analyze how FUS-P423L recognizes H3K36me3 genome-wide, we called FUS and H3K36me3 overlapping peaks in WT cells and then calculated the enrichment of FUS in WT and FUS-P423L mutant mESCs. While WT FUS was highly enriched at these peaks, FUS-P423L was largely abolished from these genomic regions (Figure 6C and D). In addition, visualization with IGV revealed that FUS was abolished at H3K36me3-enriched regions in FUS-P423L mutant mESCs (Figure 6E).

We next performed FUS RIP-seq to investigate the RNA-binding ability of the FUS-P423L protein. HeLa cells were spiked in as normalization controls. Two independent replicates were highly correlated, and the data were merged for further analysis (Supplemental Table S1). The average RNA-binding signals of FUS-P423L at all genes were higher than those of WT FUS (Figure 6F). The FUS-binding signals at each gene were the most highly increased in the FUS-P423L mutant cells (Figure 6G). In addition, the FUS-binding increased RNA species largely overlapped between the FUS-P423L mutant and *Setd2* KO mESCs (Figure 6H). Genes with increased FUS-P423L binding, regardless of H3K36me3 levels, were enriched in ribonucleoprotein complex biogenesis and metabolite generation (Supplemental Figure S7C). Out of a total of 667 genes, 63 genes exhibited changes in their APA preferences (Supplemental Figure S7D). More importantly, the increase in RNA-binding with FUS-P423L was highly correlated with the enrichment of H3K36me3 at gene bodies (*R* = 0.54) but not with the enrichment of H3K36me3 at promoter regions (*R* = 0.18), further demonstrating that the increase in FUS-P423L and RNA interaction were associated with H3K36me3 at gene bodies (Figure 6I and J). We further analyzed how the increased binding of FUS-P423L affected the APA of genes. The APA-altered genes largely overlapped with the genes associated with increased FUS binding (Figure 6K). To further analyze where this increased binding between FUS and RNA was, we performed FUS RIP qPCR in nuclear and cytoplasm, respectively. It isn’t easy to compare FUS binding affinity between nuclear and cytoplasm due to variations in the levels of RNA and FUS. We then analyzed how FUS and RNA interactions were affected among different conditions. We tested four genes that exhibited a high level of enrichment in FUS RIP-seq. IgG was used as the negative control and the ratio of purified RNA in WT cells was normalized as 1. The increased binding of FUS with these RNA occurred in the nucleus when *Setd2* was knocked out or FUS was mutated (Figure 6L). These data suggest that the P423L mutation abolishes FUS binding with H3K36me3 on chromatin and increases FUS binding with RNA in the nucleus, leading to increased distal APA selection.

### Loss of H3K36me3 recognition by FUS leads to mitochondrial hyperactivation and hyperdifferentiation

**Figure 7.**
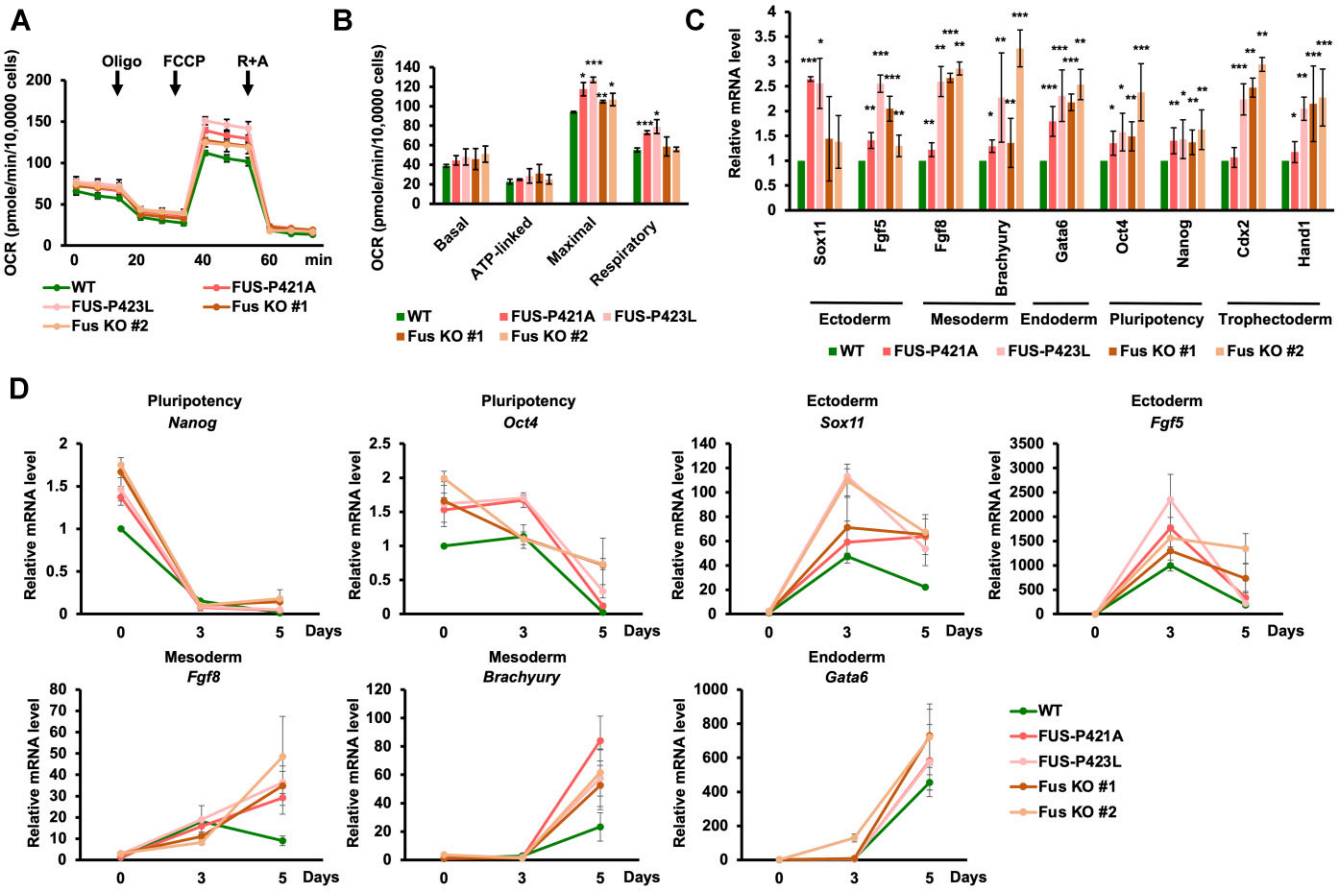
Loss of H3K36me3 recognition of FUS causes hyperactivation of mitochondria and hyperdifferentiation in mESC. (A and B) Quantification of oxygen consumption rate (OCR) of indicated mESCs treated with small molecules (**A**) and the indicated OCR parameters (**B**). The data were represented by the mean ± SD (*N* = 3 independent replicates). * *P* < 0.05 and *** *P* < 0.001, as determined by paired t-test, one-sided. (**C**) RT-qPCR analysis of the marker genes in WT, *Fus* mutant, and *Fus* KO mESCs. Gene expressions were normalized to *Actin* and the expression levels in WT cells were further normalized as 1. The data were represented by the mean ± SD (*N* = 3 independent replicates). * *P* < 0.05, ** *P* < 0.01 and *** *P* < 0.001, as determined by paired *t*-test, one-sided. (**D**) Differentiation marker genes were expressed higher in *Fus* mutant and *Fus* KO mESCs during random differentiation. mESCs were cultured as embryoid bodies to induce random differentiation. The data were represented by the mean ± SD (*N* = 3 independent replicates). *P* values as determined by paired *t*-test, one-sided were listed in Supplemental Table S4.

The genes with an increased number of distal APA in FUS-P421A and FUS-P423L mutant mESCs were all enriched in mitochondrial function-associated GO terms (Figure 5D and E). We analyzed the gene expression of mitochondrion-associated genes with increased APA, including *Atp5a1*, *Atp5g1*, *Elob* and *Mrpl12*. The expression levels of all of these genes were increased in FUS-P421A and FUS-P423L mutant mESCs (Supplemental Figure S8A). In addition, we analyzed the degradation rates of these genes and found that these genes were degraded faster in the *Fus* mutant and *Fus* KO cells (Supplemental Figure S8B). To analyze the impact of FUS mutation on the function of mitochondria, we first measured the levels of reactive oxygen species (ROS) that were produced by mitochondria. The total levels of produced ROS were elevated in *Fus* mutant and *Fus* KO cells, whereas *Setd2* and *Smyd5* KO did not affect the production of ROS (Supplemental Figure S8C and D). It is possible that *Setd2* and *Smyd5* KO changed the expression of other genes associated with mitochondrial functions. Therefore, we analyzed the oxygen consumption rate (OCR) in WT, *Fus* mutant, and *Fus* KO mESCs. The maximal OCR was increased in the *Fus* mutant and *Fus* KO cell lines, while respiratory OCR was increased only in the FUS-P421A and FUS-P423L mutant mESCs (Figure 7A and B). These data suggest that mitochondria were hyperactive in *Fus* mutant and *Fus* KO mESCs.

Because mitochondrial dysfunction has been implicated in motor neuron death and differentiation in ALS, we examined the expression levels of pluripotency, ectoderm, mesoderm, endoderm, and trophectoderm markers in WT, *Fus* mutant and *Fus* KO cells. Marker gene expression in each category was analyzed by RT−qPCR. The expression levels of the tested marker genes were increased in *Fus* mutant and *Fus* KO cells, except for *Sox11*, which was not changed in the *Fus* KO cells (Figure 7C). Moreover, we measured the degree of WT, *Fus* mutant and *Fus* KO mESC differentiation by analyzing embryoid body (EB) formation. The expression of *Nanog* and *Oct4*, which are marker genes for cell pluripotency, was decreased in all cell lines during EB formation, indicating successful differentiation of mESCs. Compared with WT cells, the expression of marker genes in ectoderm, mesoderm, and endoderm differentiation was higher in *Fus* mutant and *Fus* KO cells throughout the differentiation process (Figure 7D). Together, these data suggest that loss of H3K36me3 recognition by FUS leads to hyperactivation of mitochondria and hyperdifferentiation in mESCs, which may, at least in part, be similar to the phenotype of ALS.

### The increase of H3K36me3 restores APA choices and rescues mitochondrial dysfunction

**Figure 8.**
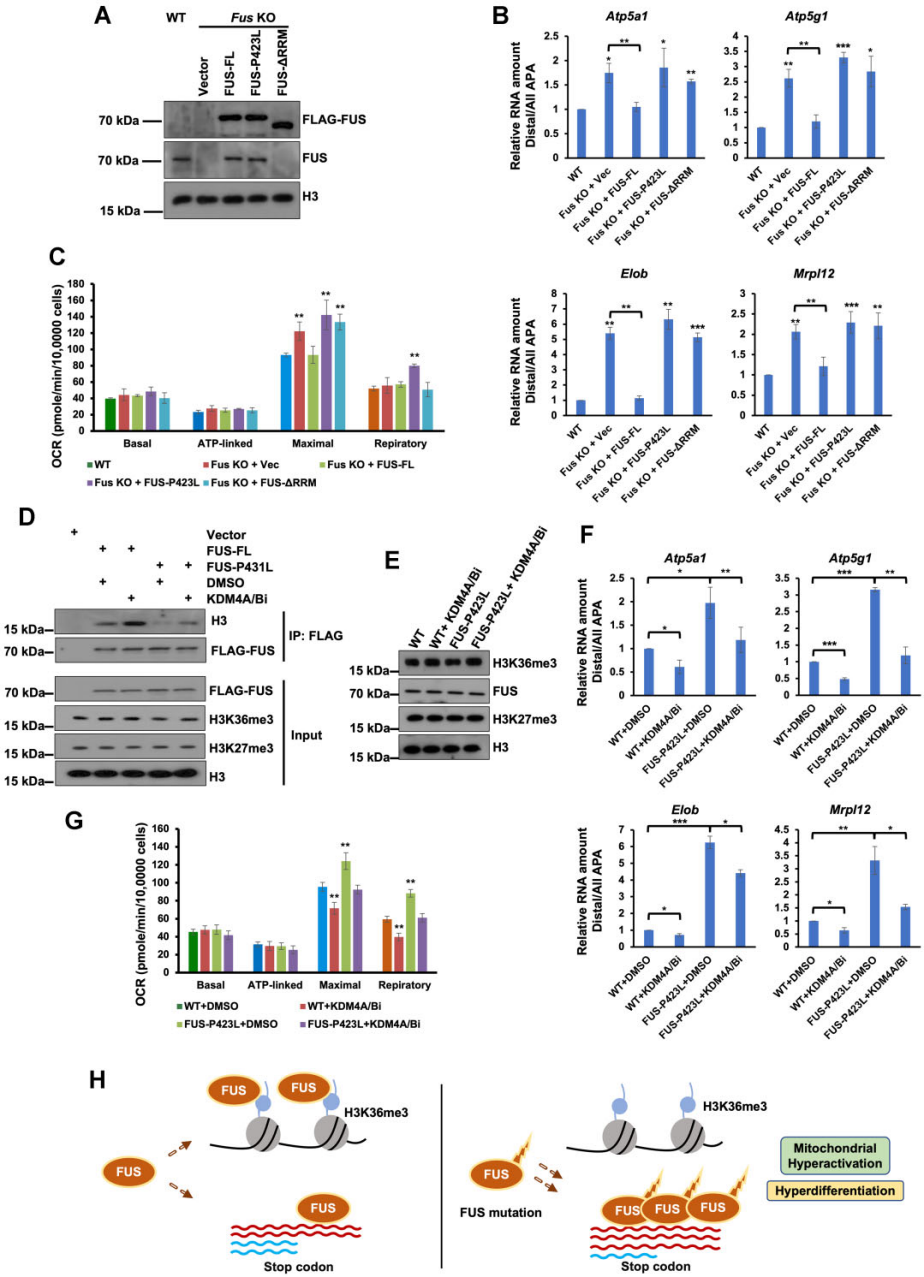
The increase in H3K36me3 helps to restore APA choices and alleviate mitochondrial dysfunction. (**A**) Western blotting result representing the total levels of indicated proteins in WT, *Fus* KO, and FUS re-expression mESCs. Full length (FL), P423L or RRM domain deleted (ΔRRM) FUS were re-expressed. Cell extracts were analyzed by Western blotting using the indicated antibodies. (**B**) RT-qPCR results showing the different polyadenylation levels of genes in WT, *Fus* KO and FUS re-expression mESCs. The ratio of distal APA in the total APA RNA in WT cells was normalized as 1. The data were represented by the mean ± SD (*N* = 3 independent replicates). ** *P* < 0.01 and *** *P* < 0.001, as determined by paired t-test, one-sided. (**C**) Quantification of oxygen consumption rate (OCR) of indicated mESCs. The data were represented by the mean ± SD (*N* = 3 independent replicates). ** *P* < 0.01 as determined by paired *t*-test, one-sided. (**D**) Treatment of KDM4A/B inhibitor (KDM4A/Bi, 10 μM NSC636819 for 48 h) increased the binding between H3 and FUS-P431L in HEK293T cells. Treatment of DMSO was used as a negative control. (**E**) Western blotting result representing the total levels of indicated proteins in WT and FUS-P423L mESCs after the treatment of DMSO or 10 μM KDM4A/B inhibitor (KDM4A/Bi, NSC636819) for 48 hours. Cell extracts were analyzed by western blotting using the indicated antibodies. (**F**) RT-qPCR results showing the different polyadenylation levels of genes in WT and FUS-P421L mESCs after the treatment of DMSO and KDM4A/B inhibitor (KDM4A/Bi, NSC636819) for 48 h, respectively. The ratio of distal APA in the total APA RNA in WT cells was normalized as 1. The data were represented by the mean ± SD (*N* = 3 independent replicates). * *P* < 0.05, ** *P* < 0.01, and *** *P* < 0.001, as determined by paired *t*-test, one-sided. (**G**) Quantification of OCR of indicated mESCs after the treatment of DMSO or KDM4A/B inhibitor (KDM4A/Bi, NSC636819) for 48 h. The data were represented by the mean ± SD (*N* = 3 independent replicates). ** *P* < 0.01 as determined by paired *t*-test, one-sided. (**H**) A model showing the function of H3K36me3 recruited FUS in APA selection.

To test whether the changes in APA were caused by FUS and RNA interaction, we overexpressed FUS-FL, FUS-P423L and FUS-ΔRRM which truncated the RNA recognition motif in *Fus* KO cells. The expression levels of overexpressed FUS and FUS mutants were similar to the endogenous FUS level. Please note the FUS antibody we used could not detect the FUS-ΔRRM likely because the antigen was designed at this region. The level of FUS-ΔRRM protein was analyzed by FLAG tag (Figure 8A). We then analyzed the choice of APA by qPCR. The results showed that FUS-FL could restore the APA choices but not the FUS-P423L or FUS-ΔRRM (Figure 8B). The expression levels of four tested mitochondrial-related genes were restored only by FUS-FL (Supplemental Figure S9A). Moreover, the total levels of produced ROS and maximal OCR were restored only in FUS-FL overexpressed cells (Figure 8C and Supplemental Figure S9B). These results indicate that the RNA binding ability is critical for the increase of proximal APA.

Since the FUS-P423L mutation reduced its H3K36me3 binding, we planned to investigate whether an increase of H3K36me3 could restore their interaction. Because the demethylases of H3K36me3 were not so specific as they may act on other H3 lysine residues, we used two complementary strategies to increase H3K36me3 in cells. One was to use a KDM4A/B inhibitor and the other was to knockdown *Ino66*. KDM4A/B inhibitor and *Ino66* shRNA increased the binding between FUS-P431L and H3 to a similar level as FUS-FL and H3 (Figure 8D and Supplemental Figure S9C). We then treated the WT and FUS-P423L mESC with KDM4A/B inhibitor and *Ino66* shRNA, respectively, resulting in an increase of H3K36me3 in cells (Figure 8E and Supplemental Figure S9D). The increase of H3K36me3 could restore the APA choices as detected by qPCR (Figure 8F and Supplemental Figure S9E). The expression levels of four analyzed mitochondrial-related genes were restored by elevated H3K36me3 (Supplemental Figure S9F and G). More importantly, the total levels of produced ROS and maximal OCR were restored in FUS-P423L mESCs (Figure 8G and Supplemental Figure S9H-J). Together, these data suggest that the increase of H3K36me3 restores APA choices and rescues mitochondrial dysfunction in the FUS-P42 mutant.

## Discussion

Histone posttranslational modifications, that is, histone marks, regulate gene expression by altering chromatin structure. Several histone marks have been reported to be correlated with gene expression (36). For example, H3K36me3, H3K4me3, H2BK120ub, and H3K79me2/me3 are associated with increased gene expression. H3K27me3 and H2AK119ub are correlated with repressed gene expression. H3K36me2, which is enriched at both the gene body and intergenic regions, is correlated with both active and repressed gene expression. However, the underlying molecular mechanism of histone marks regulation of gene expression is not yet clear. In this study, we revealed that the SETD2-mediated H3K36me3 modification modulates FUS binding to control APA in cells. The proline residues in the ZNF domain are critical for the interaction between FUS and H3K36me3. Once H3K36me3 is depleted or FUS loses the capability to recognize H3K36me3, fewer FUS molecules bind to chromatin and thus accumulate at RNA, increasing distal APA selection. Moreover, H3K36me3–FUS binding is critical for mitochondrial function and the differentiation of mESCs, which are both usually dysregulated in ALS (Figure 8H). Our findings complement the understanding of how H3K36me3 regulates gene expression.

Disorders in APA selection are estimated to be a factor in 15–50% of human diseases (74–76). In *Saccharomyces cerevisiae*, histone H3K36me3 methyltransferase Set2 controls the selection of poly(A) sites (77). Depletion of Set2 leads to elevated recruitment of the cleavage/polyadenylation complex, which changes the poly(A) sites. Set2 controls nucleosome occupancy at a poly(A) site and decreases the efficiency of poly(A) at a single site. In *Caenorhabditis elegans*, 3′ UTR length is closely correlated with the H3K36me3 level and the stability of longevity-associated mRNA expression. Inactivation of the H3K36me3 methyltransferase met-1 results in a shortened lifespan and increased gene expression changes with age (78). A similar negative correlation between mRNA expression changes and H3K36me3 enrichment has also been detected in *Drosophila melanogaster* (79). In mammalian cells, H3K36me3 forms an adaptor system with MRG15 and recruits the splicing regulator PTB to regulate RNA-splicing complex formation (80,81). Depletion of 3′ splice sites leads to a shift in H3K36me3 location, which moves toward the 3′ end, and global inhibition of splicing causes redeposition of H3K36me3. These observations suggest that H3K36me3 and cotranscriptional APA can affect each other. It would be interesting to analyze the molecular mechanism underlying how APA changes regulate H3K36me3 deposition on chromatin. H3.3S31 phosphorylation has been reported to increase SETD2 activity in the methylation of H3.3K36 (82). Whether FUS recognizes H3.3K36me3 or H3.1K36me3 is an interesting possibility to be assessed in the future.

FUS could interact with the spliceosome (21,22), transcriptional machinery (23,24) and 3′-end-processing machinery (25,26). Understanding the crucial role of FUS in APA selection is essential. The positioning of FUS binding to the poly(A) site can significantly impact the process. When FUS is bound downstream of APA, it leads to an increase in the expression of alternative short transcripts, whereas if it is bound upstream of APA, the expression of alternative short transcripts is suppressed. This is because FUS promotes polyadenylation by recruiting CPSF160 to facilitate polyadenylation when it is enriched downstream of APA, whereas when it is located upstream of APA, it blocks RNA polymerase II, leading to downregulation of the expression of alternative short transcripts (23). Proximal polyadenylation sites show higher chromatic enrichment of FUS and H3K36me3 compared to distal sites. In line with previous observations, FUS binds to the RNA to repress the proximal APA. When H3K36me3 is abolished or FUS is mutated to destroy the interaction between H3K36me3 and FUS, FUS accumulates on the RNA to repress the short transcripts, leading to increased distal APA choices. When FUS is completely knocked out, it causes a significant decrease in both FUS and RNA binding. However, we still observe an increase in distal APA choice, which seems contradict to the increased binding in FUS mutations and *Setd2* KO where the binding between FUS and RNA is increased. We speculate that, in FUS KO cells, other factors that regulate APA may play as complementarity effectors to drive a distal APA choice. The process of APA involves a protein complex composed of Cleavage and Polyadenylation Specificity Factor (CPSF), Cleavage Stimulatory Factor (CstF), and Cleavage Factor I (CFI). This complex cleaves the 3′ end of newly formed RNA, followed by polyadenylation of the cleaved end (83). RNA can directly recruit the CPSF, CstF and CFI complex (84). Additionally, phosphorylated Cytoplasmic Polyadenylation Element Binding protein (CPEB) may regulate the recruitment of CPSF, thus playing a role in the APA site choice (85). RNA Polymerase II can bind with CPSF and CstF, which enables them to signal the polymerase to terminate transcription (86). Without the regulation of FUS, other factors like RNA Polymerase II could move to the distal sites to recruit CPSF, leading to a distal APA choice. So, it is possible that other factors may contribute to the control of APA, potentially providing redundancy and complementarity by functioning alongside core factors or allowing regulation that is specific to individual genes. Without the regulation of FUS, the poly(A) sites were dysregulated, and distal APA was up-regulated. It would also be interesting to digest how the FUS KO leads to an increase in distal APA choices.

The accuracy of poly(A) length assay depends on the site of adaptor addition to mRNA. PAIso-seq employs the use of primers that anneal to mRNA and extend the mRNA with the Klenow enzyme (62). FLAM-seq uses G/I tailing to add G/I to mRNA, followed by annealing with dC-enriched primer (87). In these two methods, there is a possibility that the primers could anneal incorrectly to the poly(A) tail. So the detected length of poly(A) may be several nucleotides different. In our BOWL-seq method, when we add the oligo to anneal with mRNA, we design a ligation-based selection of primers that are perfectly annealed at the end of poly(A). The annealed primers with even a single nucleic acid shift were not ligated to mRNA, allowing for precise determination of poly(A) length.

ALS is a relentless neurodegenerative disorder characterized primarily by the loss of motor neurons, resulting in progressive impairment of the neuromuscular system (88). Mutations in more than 25 genes have been identified as pathological mutations that account for approximately 15% of ALS cases (89). FUS mutations are associated with the early onset and most aggressive forms of ALS. FUS carries a C-terminal nuclear localization signal (NLS) to guide its localization. Most FUS mutations have been found in NLS regions, causing a mislocalization of FUS and accumulation in the cytoplasm. The loss of localization to the nucleus is not sufficient to induce neurodegeneration but in conjugation with its cytoplasmic roles, the mislocalization of FUS results in cytoplasmic neuronal aggregates (32,90). Recent studies showed that mutations in the NLS regions led to liquid-liquid phase separation (LLPS) of FUS in the cytoplasm, which affected many RNA metabolic processes, including RNA splicing (33,91). Moreover, mutations in RGG domains disrupt π–π interactions between FUS proteins to regulate LLPS (92). Our results were consistent with the observations showing that proline mutations in the ZNF domain, in addition to the deletion of the ZNF domain, induced the accumulation of FUS in the cytoplasm, where it formed large aggregates (Supplemental Figure S4). Emerging evidence supports toxic gain-of-function of ALS-associated mutant FUS that leads to LLPS and subsequently abnormal assembly of dynamic ribonucleoprotein granules, which induces the neurodegenerative disorder in ALS.

ZMYND11 is an H3K36me3 reader that specifically binds H3K36me3 on H3.3 nucleosomes and regulates the elongation of Pol II. The PWWP domain in ZMYND11 forms an aromatic cage that recognizes trimethylated H3K36 and is arranged in tandem with the bromo domain to form a composite pocket that specifically recognizes the H3.3 serine 31 residue. ZMYND11 functions as an unconventional transcription corepressor that prevents hyperactivation of transcription (48–50). The tandem bromo-PWWP domain in ZMYND11, which recognizes H3K36me3, forms a V-shaped structure imparted via a ‘kink’ around a ZNF motif (48). The PWWP domains in ZMYND11 and several other proteins have also been characterized as DNA-binding domains, suggesting a dual role for the PWWP domain in recognizing DNA and H3K36me3 (93,94). This supposition is consistent with our results showing that the ZNF domain, as a DNA-binding domain in FUS, binds H3K36me3 octamers. The synergy between histone modification and DNA may contribute to H3K36me3 recognition at the nucleosome level.

FUS is an RNA-binding protein involved in multiple aspects of RNA processing. FUS binds RNA with a threefold stronger affinity than FUS binding to single-strand DNA, and FUS binding to double-strand DNA is relatively weak (69). Its RNA-binding ability has been suggested to be crucial for the function of FUS. Transcriptome studies have shown that FUS interacts with RNA harboring a variety of motifs, indicating that FUS binds RNA in a complicated way. *In vitro* SELEX analysis and *in vivo* high-throughput sequencing analysis have revealed several RNA sequences bound by the FUS protein (23,95). Both RNA sequences and secondary structures are suggested to be FUS binding signatures. Among these signatures, CGCGC, GGUG, and GUGGU are primary sequence motifs recognized by FUS (25,72,73). In addition, the stem-loop structure, which opens via a U•C or U•U non-Watson-Crick base pair, and the telomeric repeat-containing RNA (TERRA), which includes UUAGGG repeats forming a G-quadruplex, are secondary structures preferentially bound by FUS protein (96). However, these five specific sequences/motifs show similar FUS affinities, with *K*_d_^app^ values spanning a 10-fold range. Several tested RNAs without these motifs bind FUS with similar affinities as RNAs with FUS-binding sequences/motifs. FUS binds RNA in a length-dependent manner, further indicating that the affinity between RNA and FUS is not sequence-specific (69). Moreover, RNAs with these reported motifs represent less than 10% of the identified FUS-binding regions (69). These studies strongly suggest that FUS binds RNA via the regulation of other factors. Our results support the idea that H3K36me3 regulates, at least in part, the localization and enrichment of FUS at chromatin and RNA, which contributes to the regulation of interactions between FUS and RNA. Moreover, other studies have shown that aberrant APA induced by FUS depletion exhibits different signatures in cerebellar neurons, cortical neurons, motor neurons, and glial cells (30). It is possible that the enrichment of H3K36me3 differs in these cell types, leading to specialized localization of FUS to mRNA and regulation of distinct sets of genes.

We detected an increase in mitochondrion-associated gene expression and hyperactivation of mitochondria in both *Fus* KO and FUS-P421A/P423L mutant mESCs. It is possible that the dysregulation of FUS caused by depletion and mutation affects a similar group of genes and subsequent cellular phenotypes. In line with these results, FUS-P421A/P423L-mutant cells exhibited an increase in the OCR in mitochondria, whereas the OCR in *Fus*-KO cells was similar to that in WT cells. The increased levels of differentiation marker gene expression were higher in the FUS-P421A/P423L-mutant cells than in the *Fus* KO cells during EB formation.

We identified FUS as an H3K36me3 reader protein that may also interact with Pol II at chromatin. A close analysis of the interplay among H3K36me3, Pol II and FUS would likely lead to the identification of the H3K36me3 regulatory mechanism of mRNA expression in cells. While many histone modification enzymes have been identified and characterized, an increasing number of studies have been conducted to identify readers of specific histone modifications. These studies have led to discoveries of the underlying mechanism of how inheritable information passes through chromatin to RNA and subsequently regulates cell phenotype acquisition.

## Supplementary Material

gkae184_Supplemental_File

## Data Availability

The reference genome used in this study is available in the UCSC (http://genome.ucsc.edu) database under mouse reference genome mm9. The raw and processed sequencing data generated in this study have been deposited in the NCBI Gene Expression Omnibus (GEO) database under the accession code GSE212425.
