# Supplementary material for: FUS reads histone H3K36me3 to regulate alternative polyadenylation
Source: Nucleic Acids Res. 2024 Mar 18;52(10):5549–71. doi: 10.1093/nar/gkae184 (PMC11162772; doi:10.1093/nar/gkae184)
Supplement: gkae184_Supplemental_File [file gkae184_supplemental_file.pdf]

FUS reads histone H3K36me3 to regulate alternative polyadenylation

Junqi Jia[1, #], Haonan Fan[1, #], Xinyi Wan[1, #], Yuan Fang[1], Zhuoning Li[1], Yin Tang[1], Yanjun Zhang[1], Jun Huang[1], and Dong Fang[1, 2, *]

[1] Zhejiang Provincial Key Laboratory for Cancer Molecular Cell Biology, Life Sciences Institute, Zhejiang University, Hangzhou, Zhejiang 310058, China.

[2] Department of Medical Oncology, Key Laboratory of Cancer Prevention and Intervention, Ministry of Education, The Second Affiliated Hospital, Zhejiang University School of Medicine, Hangzhou, Zhejiang, China.

[#] These authors contribute equally

[*] Corresponding author: Dong Fang, Email: dfang@zju.edu.cn

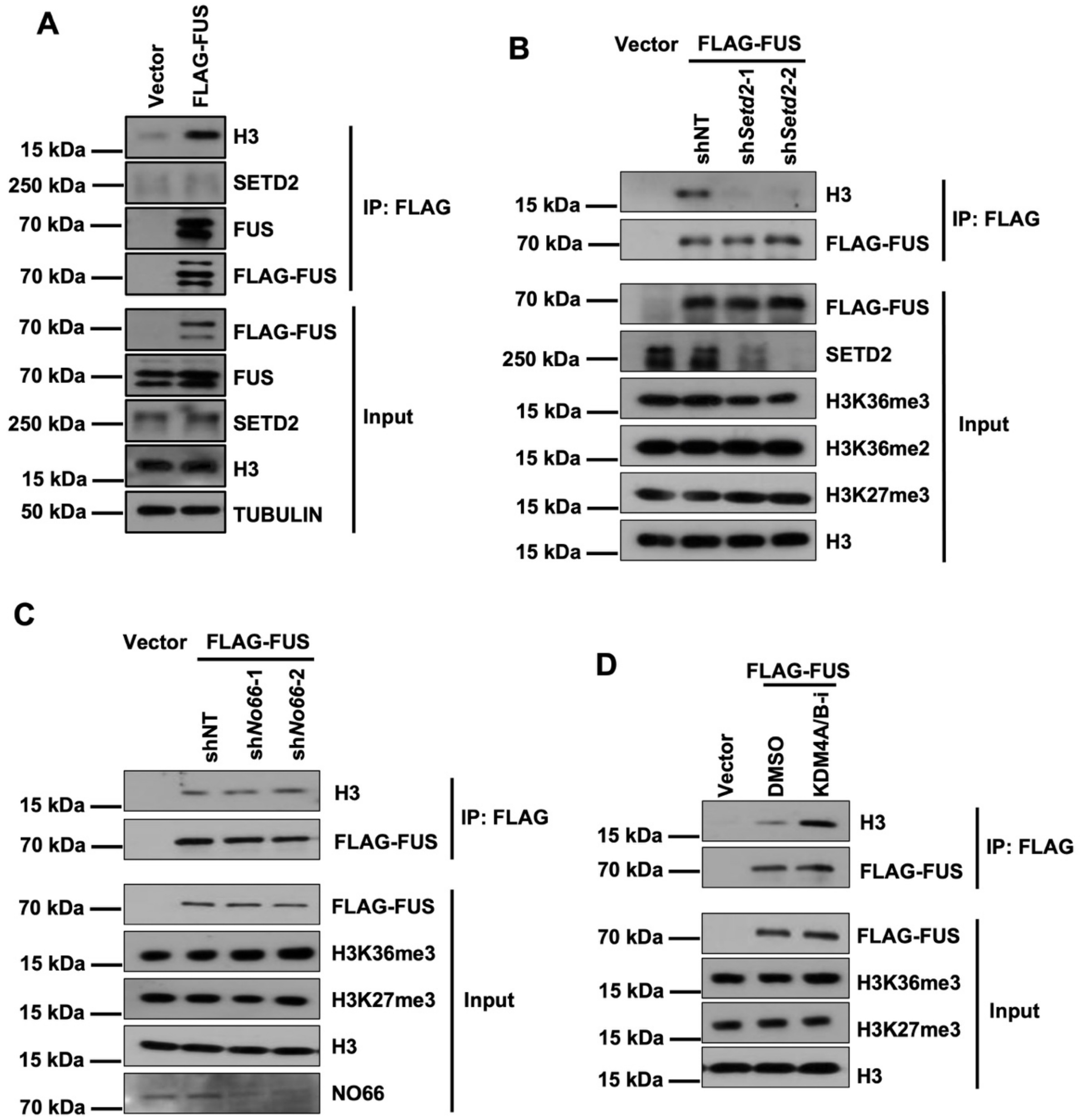

Supplemental Figure 1. FUS binds with H3K36me3.

(**A**) Overexpressed FUS bound with H3. FUS was purified by FLAG IP in HEK293T cells overexpressing FLAG-tagged FUS. HEK293T cells transfected with empty vectors were used as negative controls. Proteins from input and IP samples were analyzed by Western blotting using the indicated antibodies.

(**B**) Depletion of SETD2 decreased the binding between FUS and H3. *Setd2* was knocked down by two independent shRNA in HEK293T cells overexpressing FLAG-FUS. FLAG-FUS was purified by FLAG IP in cells. Proteins from input and IP samples were analyzed by Western blotting using the indicated antibodies. HEK293T cells that were infected with empty vectors were used as the negative control for IP experiment. NT, non-target control.

(**C**) Depletion of NO66 increased the binding between FUS and H3. *No66* was knocked down by two independent shRNA in HEK293T cells overexpressing FLAG-FUS.

(**D**) Treatment of 10 μM KDM4A/B inhibitor (KDM4A/Bi, NSC636819) for 48 hours increased the binding between FUS and H3.

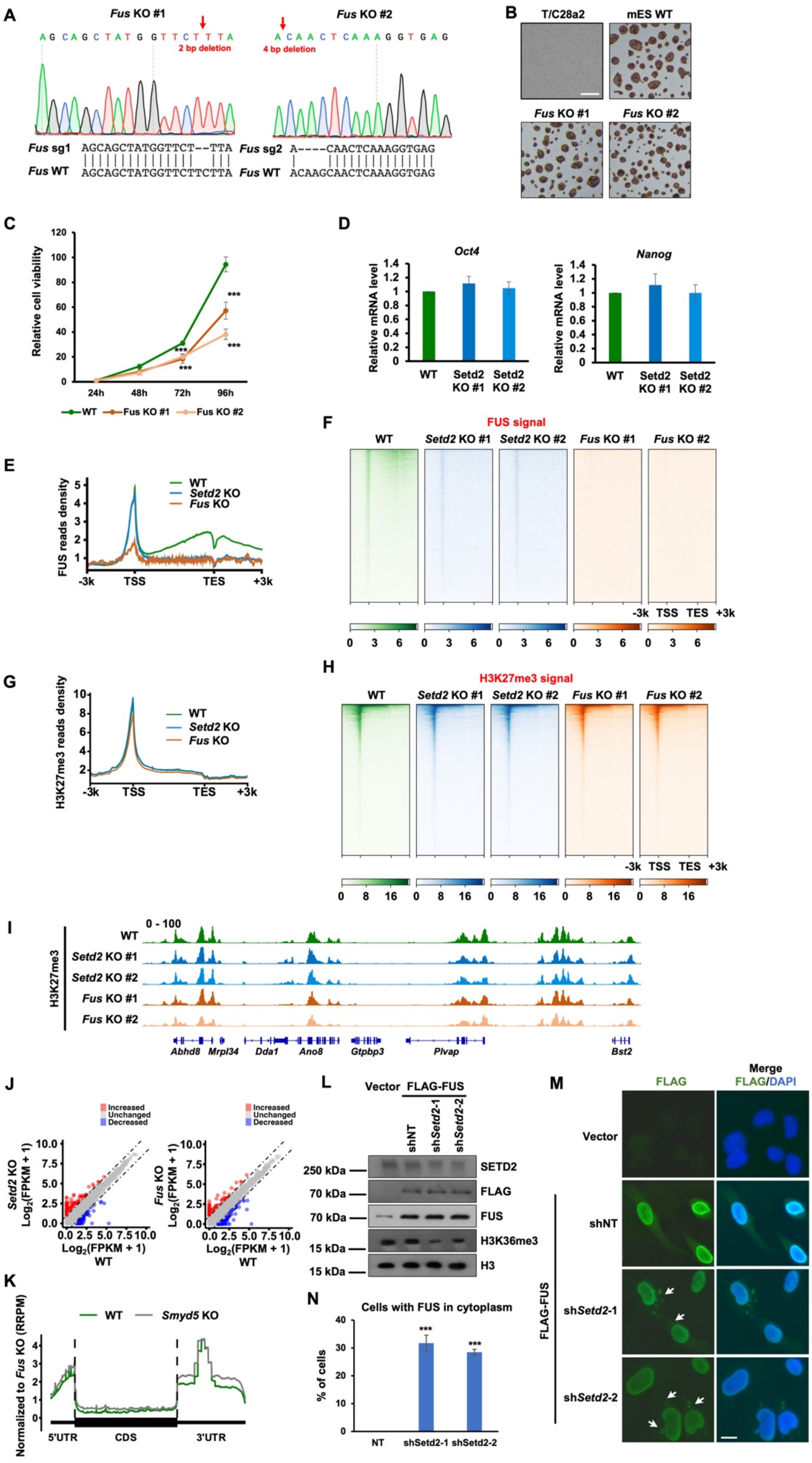

Supplemental Figure 2. ***Fus*** **KO did not affect H3K27me3 in cells.**

(**A**) Two alleles of *Fus* were mutated in *Fus* KO mESC clones. The Sanger sequencing results of the *Fus* gene locus were shown. 2 bp deletion and 4 bp deletion were identified at both alleles in *Fus* KO #1 and #2 clones, respectively.

(**B**) Alkaline phosphatase activities were preserved in *Fus* KO mESCs. T/C28a2 cells were used as negative controls. Scale bar, 200 µm.

(**C**) Cell proliferation was repressed in *Fus* KO mESCs. Data represented the mean ± SD (N = 3 independent replications). *P* value was determined by Student's t-test, one-sided. *** $p < 0.001$.

(**D**) RT-qPCR analysis of *Oct4* and *Nanog* in WT and *Setd2* KO mESCs. Gene expressions were normalized to *Actin* and the expression levels in WT cells were further normalized as 1. The data were represented by the mean ± SD (N = 3 independent replicates).

(**E**) The normalized reads distribution profiles of FUS CUT&Tag spanning 3 Kb of gene bodies in WT, *Fus* KO, and *Setd2* KO mESCs. The average read density at all genes identified by NCBI RefSeq was plotted. TSS, transcription start site. TES, transcription end site.

(**F**) Heatmaps showing FUS levels detected by H3K27me3 CUT&Tag around gene body regions in WT, *Fus* KO, and *Setd2* KO mESCs. 3 Kb windows spanning the TSS to TES of all genes determined by NCBI RefSeq were plotted. Genes were organized by their enrichments of FUS in WT cells.

(**G**) The normalized reads distribution profiles of H3K27me3 CUT&Tag spanning 3 Kb of gene bodies in WT, *Fus* KO, and *Setd2* KO mESCs. The average read density at all genes identified by NCBI RefSeq was plotted. TSS, transcription start site. TES, transcription end site.

(**H**) Heatmaps showing H3K27me3 levels detected by H3K27me3 CUT&Tag around gene body regions in WT, *Fus* KO, and *Setd2* KO mESCs. 3 Kb windows spanning the TSS to TES of all genes determined by NCBI RefSeq were plotted. Genes were organized by their enrichments of H3K27me3 in WT cells.

(**I**) IGV tracks presenting the enrichment of H3K27me3 by CUT&Tag in WT, *Fus* KO, and *Setd2* KO mESCs.

(**J**) The gene expressions in indicated mESC cell lines with spik in Hela cells as the internal control. Red dots indicate upregulated genes, blue dots indicate downregulated genes, and gray dots indicate genes with unchanged expression. Red:194, gray:24906, blue:109 in *Setd2* KO. Red:123, gray:24939, blue:147 in *Fus* KO. FPKM, fragments per kilobase of transcript per million mapped reads.

(**K**) The normalized read distribution profiles of FUS RIP-seq signals spanning gene bodies in WT and *Smyd5* KO mESCs. The average read density at all genes identified by NCBI RefSeq was plotted. RIP-seq signals were normalized by the spiked-in Hela cells first and then normalized by the signals in *Fus* KO cells. UTR, untranslated regions. CDS, coding sequence. RRPM, reference-adjusted reads per million.

(**L**) Western blotting result representing the total levels of indicated proteins in WT and *Setd2* KD HeLa cells. Cell extracts were analyzed by Western blotting using the indicated antibodies. NT, non-target control.

(**M**) IF results showing the localization of FUS after depletion of SETD2. *Setd2* was knocked down by two independent shRNA in HeLa cells overexpressing FLAG-FUS. FLAG antibody was used to stain FLAG-tagged FUS. DAPI was used to stain DNA in the nucleus. Scale bar, 50 μm. NT, non-target control. Arrows indicate FUS signals in the cytoplasm.

(**N**) The quantification results of cells with FUS in cytoplasm. The data were represented by the mean ± SD (N = 3 independent replicates). *** $p < 0.001$, as determined by paired t-test, one-sided. Over 50 cells were counted in each treatment group.

A

Sample name	Unique CCS	CCS assigned to genes	Genes recovered
WT	112530	64825	6416
Setd2 KO #1	89265	35776	5226
Setd2 KO #2	124556	73854	7055
Fus KO #1	60789	40497	5382
Fus KO #2	83139	43403	5693

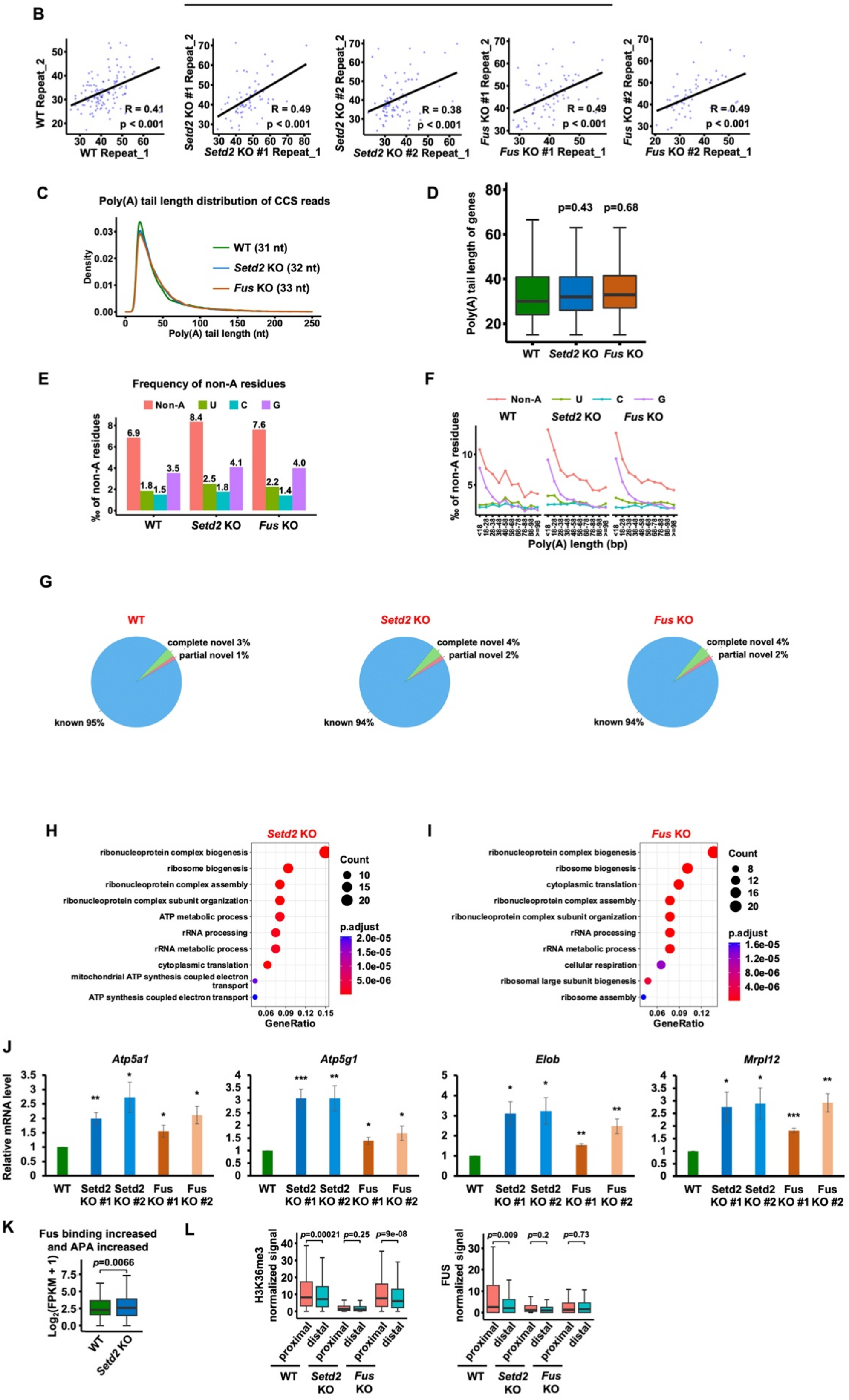

Supplemental Figure 3. APA was altered in *Setd2* and *Fus* KO mESCs.

(**A**) Reads counts in different Bowl-seq libraries.

(**B**) The correlation of gene poly(A) tail length among each cell line. R was calculated as Pearson's correlation coefficient.

(**C**) Global distribution of poly(A) tail lengths of all CCS. The median poly(A) tail length of CCS reads was shown.

(**D**) Box plot showing each gene's median poly(A) tail length. *P* value was determined by Student's t-test, two-sided.

(**E**) The percentage of non-A residues in the poly(A) tail in WT and *Fus* KO mESCs.

(**F**) The distribution of non-A residues in the poly(A) tail in WT and *Fus* KO mESCs.

(**G**) The quantification of RNA splicing events in different cell lines as determined by RNA-seq. The annotation of the splice junction in different RNA-seq data sets was indicated in the figure. The genes from Ensembl, UCSC, and RefSeq were combined and used as a reference gene model.

(**H** and **I**) GO terms showing the genes with increased distal APA selection in *Setd2* KO mESCs (H) and *Fus* KO mESCs (I).

(**J**) RT-PCR results showing the expression levels of genes. Gene expression was normalized to *Actin* and three repeats were performed. The data were represented by the mean ± SD (N = 3 independent replicates). * $p < 0.05$, ** $p < 0.01$, and *** $p < 0.001$, as determined by paired t-test, one-sided.

(**K**) Expression levels of genes with increased APA and FUS binding in the indicated mESCs. P value was determined by unpaired t-test, one-sided.

(**L**) The reads density at proximal and distal APA in APA altered genes. P value was determined by paired t-test, two-sided.

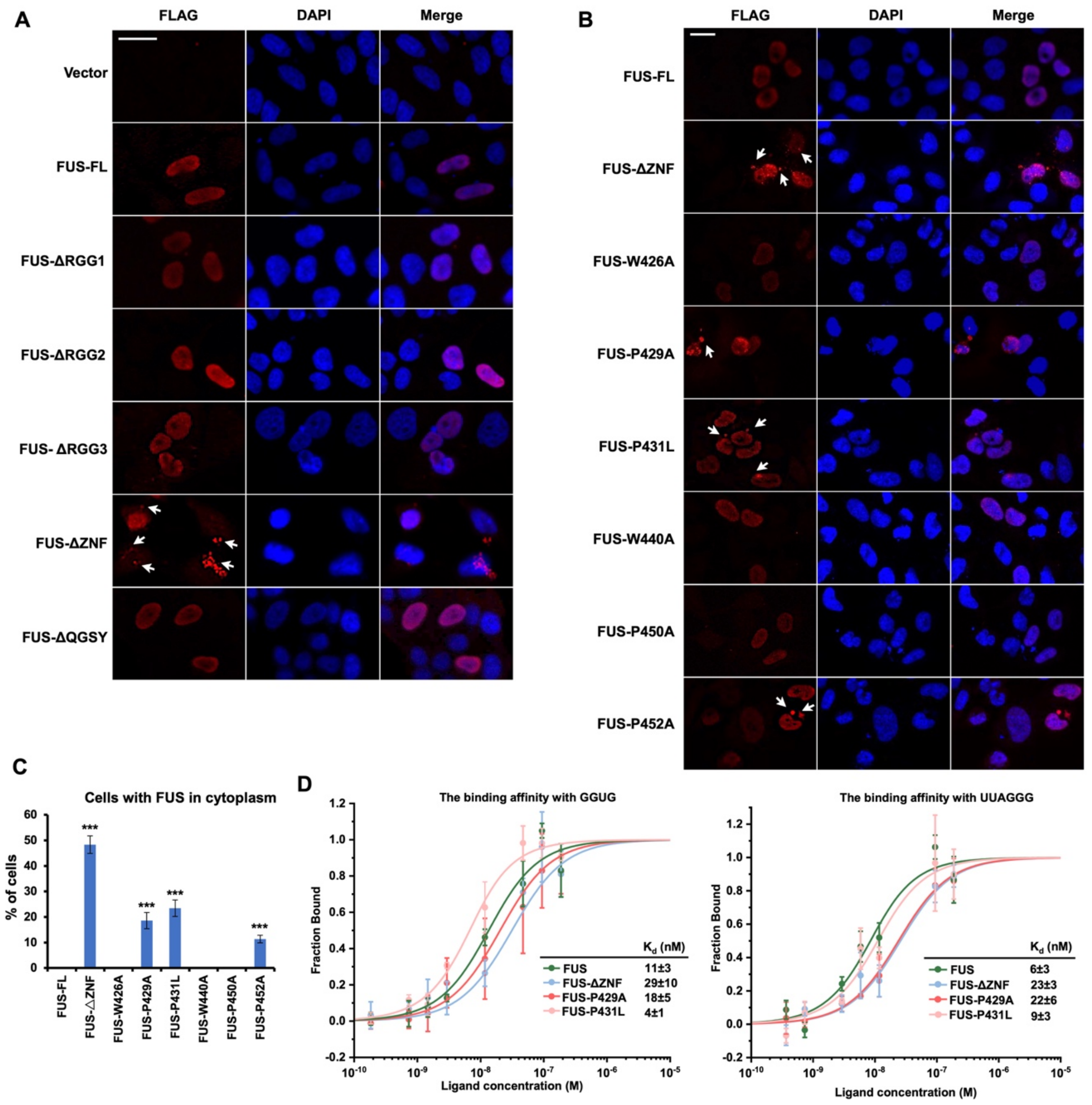

Supplemental Figure 4. FUS accumulated in the cytoplasm when lost the binding with H3.

(**A**) IF results showing the localization of WT and mutant FUS. FLAG-tagged FUS were overexpressed in Hela cells and subjected to staining with FLAG antibodies. DAPI was used to stain the DNA in nucleus. Arrows indicated FUS in the cytoplasm. Scale bar, 24 μm.

(**B**) Same as in (A), except different mutations of FUS were overexpressed.

(**C**) The quantification results of cells with FUS in cytoplasm. The data were represented by the mean ± SD (N = 3 independent replicates). *** $p < 0.001$, as determined by paired t-test, one-sided. Over 50 cells were counted in each treatment group.

(**D**) Binding curves were plotted for FUS and RNAs. The data were represented by the mean ± SD (N = 3 independent replicates).

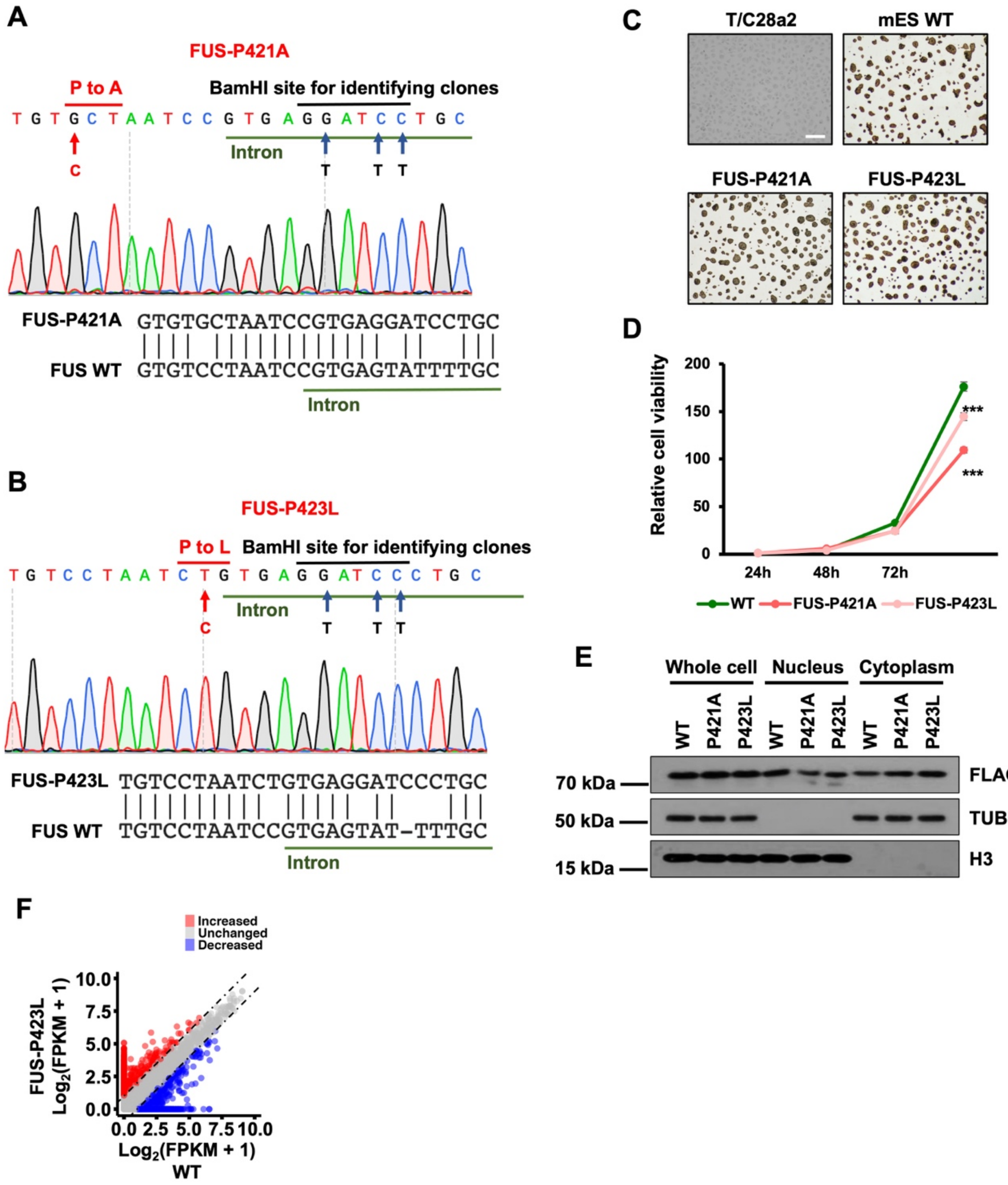

Supplemental Figure 5. Proline mutations of FUS affected the localization of FUS.

(**A**) Two alleles of *Fus* were mutated to FUS-P421A in the mESC clone. The Sanger sequencing result of the *Fus* gene locus was shown. Three bps in the intron of *Fus* were mutated to delete the sgRNA targeting and generate a BamHI cutting site for identifying mutant clones.

(**B**) Same as in (A), except two alleles of *Fus* were mutated to FUS-P423L.

(**C**) Alkaline phosphatase activities were preserved in *Fus* mutant mESCs. T/C28a2 cells were used as negative controls. Scale bar, 200 µm.

(**D**) Cell proliferation was repressed in *Fus* mutant mESCs. Data represented the mean ± SD (N = 3 independent replications). *P* value was determined by Student's t-test, one-sided. *** $p < 0.001$.
(**E**) FUS-P421A and FUS-P423L mutant proteins were decreased in the nucleus and increased in the cytoplasm. The whole-cell lysate, nuclear fraction, and cytoplasmic fraction from parental and FUS mutant-expressed HEK293T cells were analyzed by the indicated antibodies. TUBULIN which was in the cytoplasmic fraction was used as the control for the cytoplasmic fraction. H3 was used as the control for the nuclear fraction.
(**F**) The gene expressions in indicated mESC cell lines with spik in Hela cells as the internal control. Red dots indicate upregulated genes, blue dots indicate downregulated genes, and gray dots indicate genes with unchanged expression. Red:1026, gray:23004, blue:1179. FPKM, fragments per kilobase of transcript per million mapped reads.

A

Sample name	Unique CCS	CCS assigned to genes	Genes recovered
WT	105786	64150	6369
FUS-P421A	109147	75270	6949
FUS-P423L	100034	56561	6468

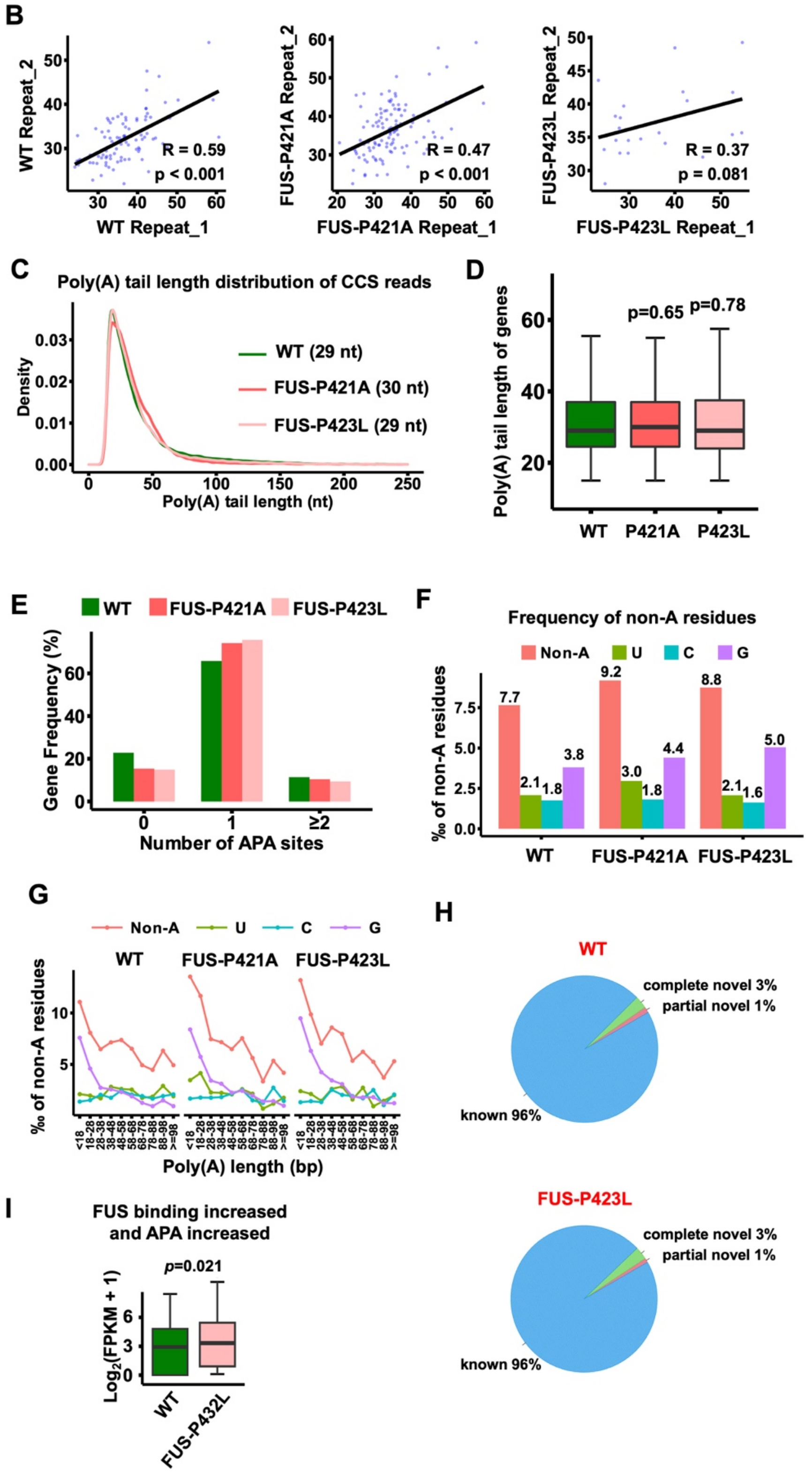

Supplemental Figure 6. APA was altered in FUS mutant mESCs.

(**A**) Reads counts in different Bowl-seq libraries.

(**B**) The correlation of gene poly(A) tail length among each cell line. R was calculated as Pearson's correlation coefficient.

(**C**) Global distribution of poly(A) tail lengths of all CCS. The median poly(A) tail length of CCS reads was shown.

(**D**) Box plot showing each gene's median poly(A) tail length. *P* value was determined by Student's t-test, two-sided.

(E) APA events of transcripts detected by Bowl-seq. APA site = 0 presented genes that were not detected with a defined poly(A) site.

(**F**) The percentage of non-A residues in the poly(A) tail in WT, FUS-P421A, and FUS-P423L mESCs.

(**G**) The distribution of non-A residues in the poly(A) tail in WT, FUS-P421A, and FUS-P423L mESCs.

(**H**) The quantification of RNA splicing events in indicated cell lines as determined by RNA-seq. The annotation of the splice junction in different RNA-seq data sets was indicated in the figure. The genes from Ensembl, UCSC, and RefSeq were combined and used as a reference gene model.

(**I**) Expression levels of genes with increased APA and FUS binding in the indicated mESCs. The p value was determined by unpaired t-test, one-sided.

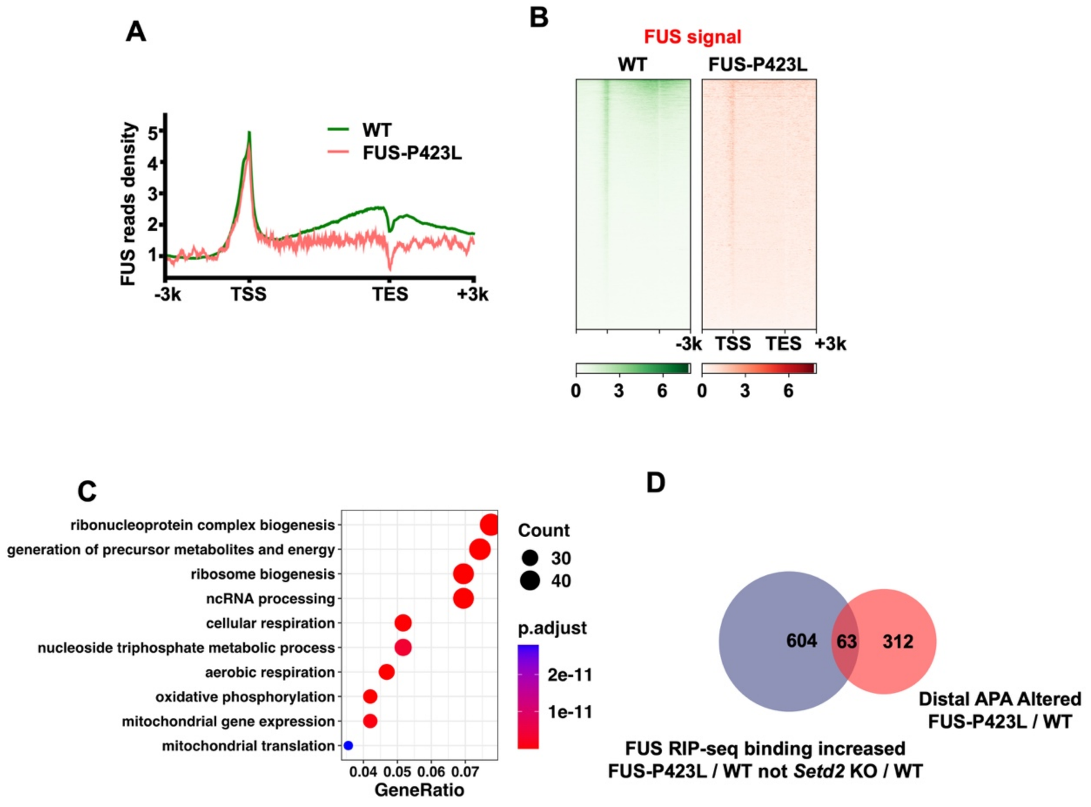

Supplemental Figure 7. FUS-P421L mutation was not enriched at gene bodies.

(**A**) The normalized reads distribution profiles of FUS CUT&Tag spanning 3 Kb of gene bodies in WT and FUS-P423L mESCs. The average read density at all genes identified by NCBI RefSeq was plotted. TSS, transcription start site. TES, transcription end site.

(**B**) Heatmaps showing FUS levels detected by FUS CUT&Tag around gene body regions in WT and FUS-P423L mESCs. 3 Kb windows spanning the TSS to TES of all genes determined by NCBI RefSeq were plotted. Genes were organized by their enrichments of FUS in WT cells.

(**C**) GO terms showing the genes with increased FUS binding in FUS-P423L but not in *Setd2* KO mESCs.

(**D**) Venn diagram showing the APA altered genes in FUS-P423L mESCs overlapping with genes, which showed increased FUS binding in FUS-P423L but not in *Setd2* KO mESCs.

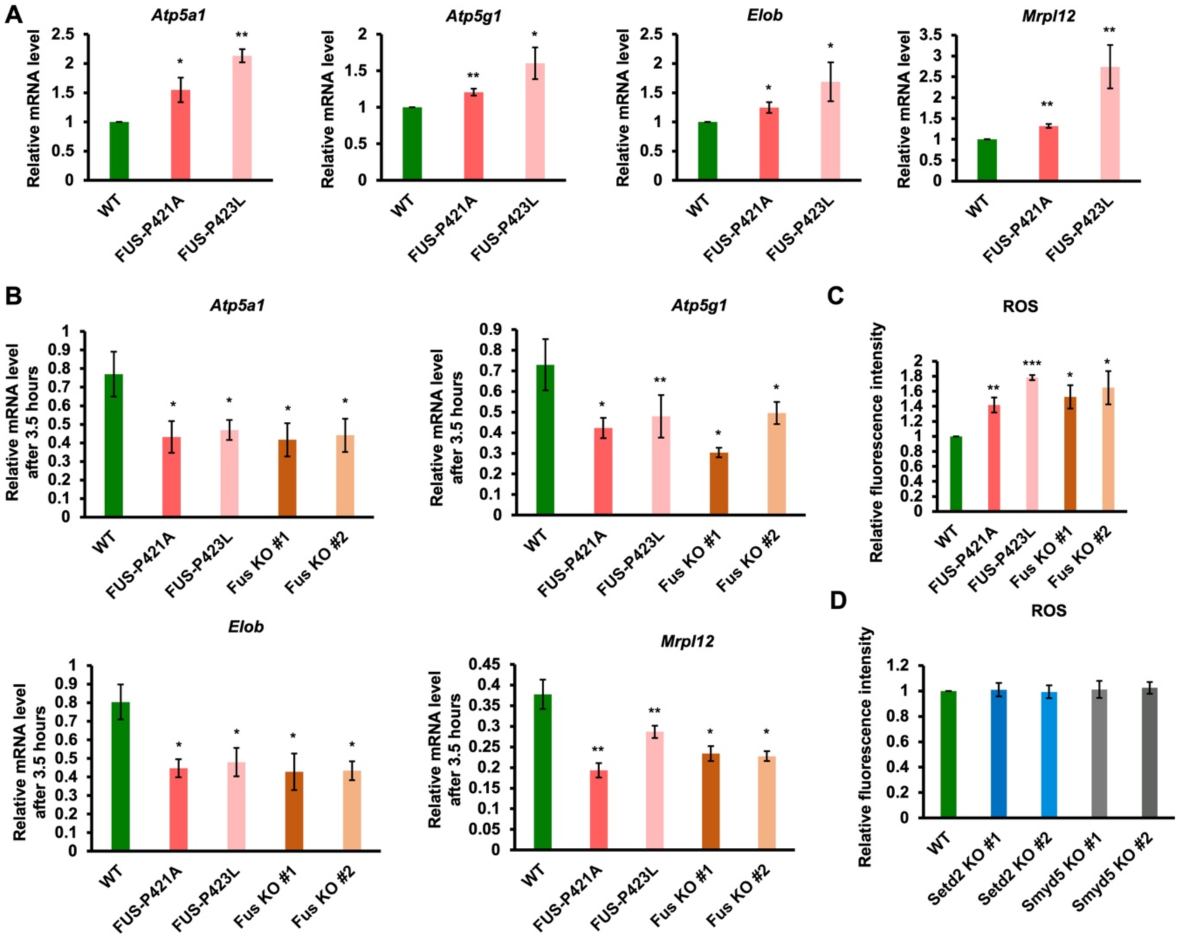

Supplemental Figure 8. FUS mutations increased the ROS in mESCs.

(**A**) RT-qPCR analysis of the mitochondrial-associated genes in WT and *Fus* mutant mESCs. Gene expressions were normalized to *Actin* and the expression levels in WT cells were further normalized as 1. The data were represented by the mean ± SD (N = 3 independent replicates). * $p < 0.05$, ** $p < 0.01$, as determined by paired t-test, one-sided.

(**B**) RT-qPCR analysis of relative mRNA levels after the treatment of actinomycin D. Total RNA before and after the treatment of actinomycin D for 3.5 hours was collected. Human RNA was spiked in before the reverse transcription. Gene expressions were normalized to human *Actin* and the expression levels before actinomycin D treatment were further normalized as 1. The relative mRNA levels were then calculated as compared to the levels before actinomycin D treatment, respectively. The data were represented by the mean ± SD (N = 3 independent replicates). * $p < 0.05$, ** $p < 0.01$, as determined by paired t-test, one-sided.

(**C**) The quantification of the ROS levels in WT, *Fus* mutant, and *Fus* KO mESCs. The data were represented by the mean ± SD (N = 3 independent replicates). * $p < 0.05$, ** $p < 0.01$ and *** $p < 0.001$, as determined by paired t-test, one-sided.

(**D**) Same as in (C), except ROS levels in WT, *Setd2* KO, and *Smyd5* KO mESCs were analyzed.

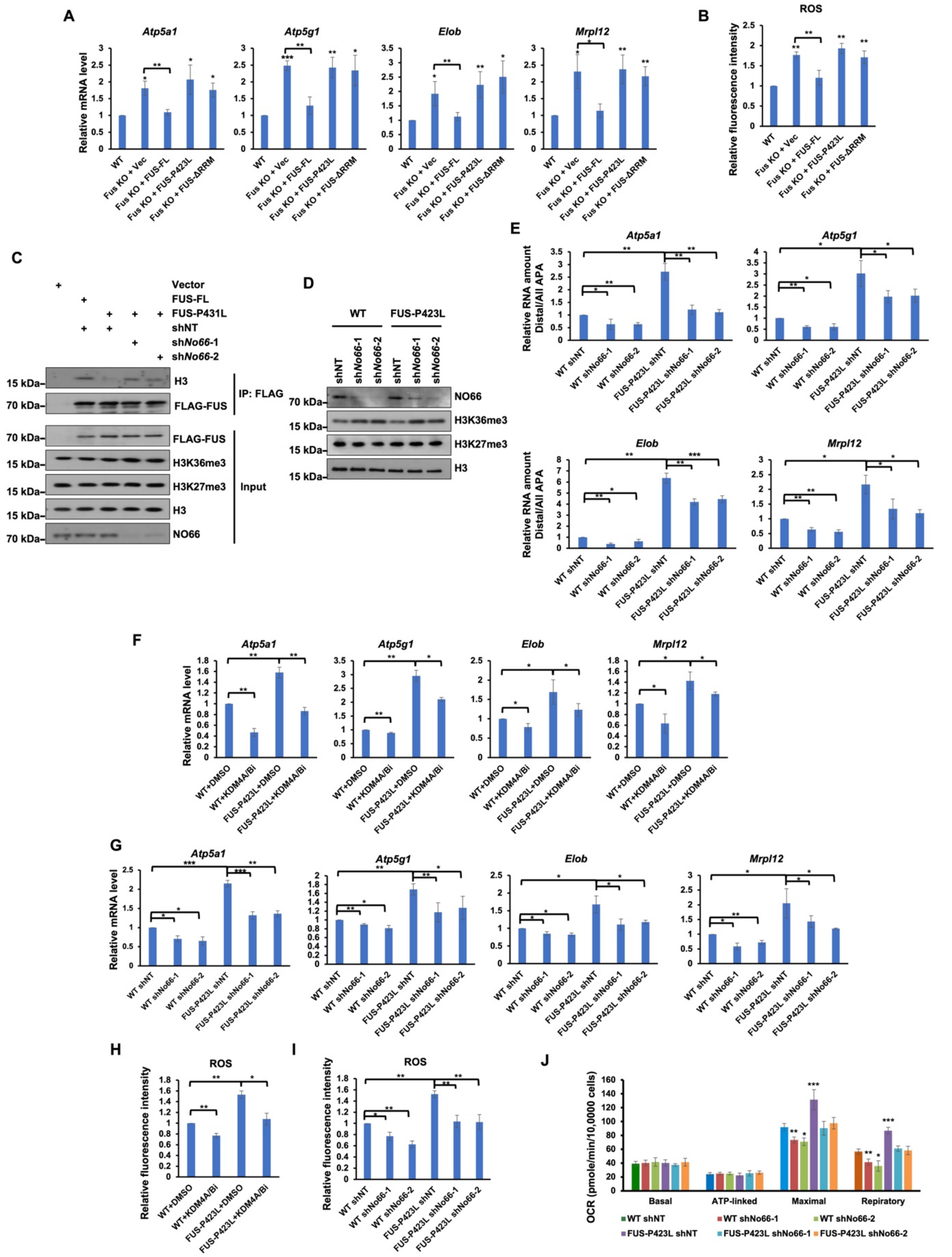

Supplemental Figure 9. Elevated H3K36me3 rescued the APA and mitochondrial function in FUS-P423L mESCs.

(**A**) RT-qPCR analysis of the mitochondrial function-associated genes in WT, *Fus* KO, and FUS re-expression mESCs. Full length (FL), P423L, or RRM domain deleted (ΔRRM) FUS

were re-expressed. Gene expressions were normalized to *Actin* and the expression levels in WT cells were further normalized as 1. The data were represented by the mean ± SD (N = 3 independent replicates). * $p < 0.05$, ** $p < 0.01$, and *** $p < 0.001$,as determined by paired t-test, one-sided.

(**B**) The quantification of the ROS levels in WT, *Fus* KO, and FUS re-expression mESCs. ** $p < 0.01$ as determined by paired t-test, one-sided.

(**C**) Depletion of NO66 restored the binding between FUS-P431L and H3. *No66* was knocked down by two independent shRNA in HEK293T cells overexpressing FUS-WT and FUS-P431L, respectively. FLAG-FUS was purified by FLAG IP in cells. Proteins from input and IP samples were analyzed by Western blotting using the indicated antibodies. HEK293T cells that were infected with empty vectors were used as the negative control for IP experiment. NT, non-target control.

(**D**) Western blotting result representing the total levels of indicated proteins in WT and FUS-P423L mESCs after depletion of NO66. Cell extracts were analyzed by Western blotting using the indicated antibodies.

(**E**) RT-qPCR results showing the different polyadenylation levels of genes in WT and FUS-P423L mESCs after depletion of NO66 or control. The ratio of distal APA in the total APA RNA in WT cells was normalized as 1. The data were represented by the mean ± SD (N = 3 independent replicates). * $p < 0.05$, ** $p < 0.01$, and *** $p < 0.001$, as determined by paired t-test, one-sided.

(**F**) RT-qPCR analysis of the mitochondrial function-associated genes in WT and FUS-P423L mESCs after the treatment of DMSO or KDM4A/B inhibitor (KDM4A/Bi, NSC636819) for 48 hours. Gene expressions were normalized to *Actin* and the expression levels in WT cells were further normalized as 1. The data were represented by the mean ± SD (N = 3 independent replicates). * $p < 0.05$, ** $p < 0.01$, as determined by paired t-test, one-sided.

(**G**) Same as in (F), except cells with NO66 depletion were analyzed.

(**H**) Quantification of ROS in indicated mESCs after the treatment of DMSO or KDM4A/B inhibitor (KDM4A/Bi, NSC636819) for 48 hours. The data were represented by the mean ± SD (N = 3 independent replicates). * $p < 0.05$, ** $p < 0.01$, and *** $p < 0.001$, as determined by paired t-test, one-sided.

(**I**) Same as in (H), except cells with NO66 depletion were analyzed.

(**J**) Quantification of OCR of indicated mESCs after depletion of NO66. The data were represented by the mean ± SD (N = 3 independent replicates). *** $p < 0.001$ as determined by paired t-test, one-sided.

Supplemental Table S1. Correlations between two replicates of epigenomic sequencing results. A 1 kb sliding window across the whole genome was used to calculate the Pearson product moment correlation for epigenomic sequencing.

Samples	Correlation
Fus KO #1 IP RIP-seq	0.9
Fus KO #1 Input RIP-seq	0.9
Fus KO #2 IP RIP-seq	0.9
Fus KO #2 Input RIP-seq	0.9
Setd2 KO #1 IP RIP-seq	0.9
Setd2 KO #1 Input RIP-seq	0.99
Setd2 KO #2 IP RIP-seq	0.9
Setd2 KO #2 Input RIP-seq	0.9
WT IP RIP-seq	0.9
WT Input RIP-seq	0.9
FUS-P423L IP RIP-seq	0.9
FUS-P423L Input RIP-seq	0.9
Smyd5 KO IP RIP-seq	0.9
Smyd5 KO Input RIP-seq	0.9
WT IP RIP-seq	0.9
WT Input RIP-seq	0.9
FUS_*Fus*_KO#1	0.5
FUS_*Fus*_KO#2	0.4
FUS_*Setd2*_KO#1	0.8
FUS_*Setd2*_KO#2	0.7
FUS_WT	0.8
H3K27me3_*Setd2*_KO#1	0.9
H3K27me3_*Setd2*_KO#2	0.9
H3K27me3_WT	0.9
H3K36me3_*Setd2*_KO#1	0.7
H3K36me3_*Setd2*_KO#2	0.8
H3K36me3_WT	0.8

Supplemental Table S2. Oligonucleotides used in the study.

Name	5'- 3'	Application
*Fus*_m_sgRNA1	AGCAGCTATGGTTCTTCTTA	*Fus* KO
*Fus*_m_sgRNA2	CTCACCTTTGAGTTGCTTGT	*Fus* KO
*Fus*_m_sgRNA3	AAGCAAAATACTCACGGATT	FUS Knock-in
β-actin_m_RT_F	GTGACGTTGACATCCGTAAAGA	Gene expression
β-actin_m_RT_R	GCCGGACTCATCGTACTCC	Gene expression
*Atp5a1*_m_RT_F	GAGACTGGGCGTGTGTTAAG	Gene expression
*Atp5a1*_m_RT_R	CTCCTCTGCTTGAACATTCCTC	Gene expression
*Cct5*_m_RT_F	GGCGATGTGACTATAACAAACGA	Gene expression
*Cct5*_m_RT_R	AGCAATTCTGATTGGGTGGATG	Gene expression
*Atp5g1*_m_RT_F	CCAGAGGCCCCATCTAAGC	Gene expression
*Atp5g1*_m_RT_R	CCCCAGAATGGCATAGGAGAAG	Gene expression
*Elob*_m_RT_F	AGCAGCGGCTTTACAAGGATG	Gene expression
Elob _m_RT_R	CACACTCGCCCAGAGTTTTG	Gene expression
*Mrpl12*_m_RT_F	ATCTCAGACCTCAACGAACTCC	Gene expression
*Mrpl12*_m_RT_R	AGTGTGTCCGCTCTTTCTGTT	Gene expression
*Sox11*_m_RT_F	CGACGACCTCATGTTCGACC	Gene expression
*Sox11*_m_RT_R	GACAGGGATAGGTTCCCCG	Gene expression
*Fgf5*_m_RT_F	AGCGCGACGTTTTCTTCGT	Gene expression
*Fgf5*_m_RT_R	GCCATTGACTTTGCCATCCG	Gene expression
*Fgf8*_m_RT_F	GCTCCGGGGCCCAAGTCCTCTG	Gene expression
*Fgf8*_m_RT_R	CCGCGGGGCCACCACACC	Gene expression

**Name**	**5'- 3'**	**Application**
*Brachyury*_m_RT_F	TCCCGGTGCTGAAGGTAAATGTGT	Gene expression
*Brachyury*_m_RT_R	TTGGGCGAGTCTGGGTGGATGTAG	Gene expression
*Gata6*_m_RT_F	TGCCTCGACCACTTGCTATGAAAA	Gene expression
*Gata6*_m_RT_R	CACTGATGCCCCTACCCCTGAG	Gene expression
*Oct4*_m_RT_F	CCCGAAGCCCTCCCTACAGCAGAT	Gene expression
*Oct4*_m_RT_R	TGGGGGCAGAGGAAAGGATACAGC	Gene expression
*Nanog*_m_RT_F	CCTTCCCTCGCCATCACACT	Gene expression
*Nanog*_m_RT_R	AGAGGAAGGGCGAGGAGAGG	Gene expression
*Cdx2*_m_RT_F	GCGGCTGGAGCTGGAGAAGGAGTT	Gene expression
*Cdx2*_m_RT_R	CGGCGGCTGTGGAGGCTGTTGT	Gene expression
*Hand1*_m_RT_F	ATGTGCCCGCCGACACCAAG	Gene expression
*Hand1*_m_RT_R	CGGGCTGCTGAGGCAACTCC	Gene expression
*ActinB*_h-RT_F	CATGTACGTTGCTATCCAGGC	Gene expression
*ActinB*_h-RT_R	CTCCTTAATGTCACGCACGAT	Gene expression
HSP90ab1-F	AAACAAGGAGATTTTCCTCCGC	Gene expression
HSP90ab1-R	CCGTCAGGCTCTCATATCGAAT	Gene expression
Ptma-F	CTCTCGCCAGAGTCCTCGAA	Gene expression
Ptma-R	GGAGCTGGTATCCACTGCC	Gene expression
Eef1a1-F	ACACGTAGATTCCGGCAAGTC	Gene expression
Eef1a1-R	GATGGTTCGCTTGTCGATTCC	Gene expression
Dppa5a-F	ATGATGGTGACCCTCGTGAC	Gene expression
Dppa5a-R	ACCTCGATAAGTTCTTCGGGAG	Gene expression

Name	5'- 3'	Application
RT-primer	AAGCAGTGGTATCAACGCAGAGTA TTTTTTTTTTTTTTTTTTTVN	RT
APA-check-R	AAGCAGTGGTATCAACGCAGAGTA	
Atp5a1-F1	AGCCCTGTCACTGTCACCAGAT	
Atp5a1-F2	AGCACCATTTGTAAAGGGTTACTC	
Atp5g1-F1	CTGGTGCTGGAGTCTACTGAG	
Atp5g1-F2	CAACAACAACGTTTCTCTAAA	
Elob-F1	CCTACCCCCTAGAGACCCATT	
Elob-F2	TCTGTTCCCTCTTGTTCACAGAA	
Mrpl12-F1	GGCAGTGACAACCTTTGCAG	
Mrpl12-F2	GCTGGGAGTATAAAGGACCACC	
Single-strand DNA for FUS-P421A	GGATTCCCCAGTGGAGGTGGTGGAG GTGGAGGACAGCAACGAGCTGGAGA CTGGAAGTGTGCTAATCCGTGAGGA TCCTGCTTTTTTTAAAAAGTTCTTGTG TGGTGTTAATATTTTAAGT	FUS Knock-in
Single-strand DNA for FUS-P423L	CCCAGTGGAGGTGGTGGAGGTGGAG GACAGCAACGAGCTGGAGACTGGAA GTGTCCTAATCTGTGAGGATCCTGCT TTTTTTAAAAAGTTCTTGTGTGGTGT TAATATTTTAAGTGTTTGG	FUS Knock-in
GFP-F	AAGCAGTGGTATCAACGCAGAGTAC ATGGTGAGCAAGGGCGAGGAG	Poly(A) analysis in Bowl-seq
GFP-0A-R	AAGCAGTGGTATCAACGCAGAGTAC GGCTACCTTGTACAGCTCGTCC	Poly(A) analysis in Bowl-seq

Name	5’- 3’	Application
GFP-10A-R	AAGCAGTGGTATCAACGCAGAGTAC<br>CTTGTATTTTTTTTTTCTTGTACAGCT<br>CGTCC	Poly(A) analysis in Bowl-seq
GFP-30A-R	AAGCAGTGGTATCAACGCAGAGTAC<br>AGTCAATTTTTTTTTTTTTTTTTTTTTT<br>TTTTTTTTCTTGTACAGCTCGTCC	Poly(A) analysis in Bowl-seq
GFP-50A-R	AAGCAGTGGTATCAACGCAGAGTAC<br>AGTTCCTTTTTTTTTTTTTTTTTTTTTT<br>TTTTTTTTTTTTTTTTTTTTTTTTTTTT<br>CTTGTACAGCTCGTCC	Poly(A) analysis in Bowl-seq
GFP-70A-R	AAGCAGTGGTATCAACGCAGAGTAC<br>ATGTCATTTTTTTTTTTTTTTTTTTTTT<br>TTTTTTTTTTTTTTTTTTTTTTTTTTTTT<br>TTTTTTTTTTTTTTTTTTTCTTGTACAG<br>CTCGTCC	Poly(A) analysis in Bowl-seq
GFP-90A-R	AAGCAGTGGTATCAACGCAGAGTAC<br>CCGTCCTTTTTTTTTTTTTTTTTTTTTT<br>TTTTTTTTTTTTTTTTTTTTTTTTTTTTT<br>TTTTTTTTTTTTTTTTTTTTTTTTTTTTT<br>TTTTTTTTTTCTTGTACAGCTCGTCC	Poly(A) analysis in Bowl-seq

Supplemental Table S3. Sequencing indexes used for Bowl-seq in the study.

Index	Sequence
1	AACGTGAT
2	AAACATCG
3	ATGCCTAA
4	AGTGGTCA
5	ACCACTGT
6	ACATTGGC
7	CAGATCTG
8	CATCAAGT
9	CGCTGATC
10	ACAAGCTA

Supplemental Table S4. P values in Figure 7D.

	Day3 FUS-P421A	Day3 FUS-P423L	Day3 *Fus* KO #1	Day3 *Fus* KO #2	Day5 FUS-P421A	Day5 FUS-P423L	Day5 *Fus* KO #1	Day5 *Fus* KO #2
**FGF5**	**0.027**	**0.030**	**0.142**	**0.016**	**0.211**	**0.224**	**0.091**	**0.014**
**SOX11**	**0.148**	**0.001**	**0.132**	**0.005**	**0.021**	**0.096**	**0.035**	**0.034**
**FGF8**	**0.160**	**0.426**	**0.005**	**0.001**	**0.014**	**0.005**	**0.015**	**0.001**
**BRACHYURY**	**0.004**	**0.090**	**0.020**	**0.028**	**0.023**	**0.038**	**0.149**	**0.025**
**GATA6**	**0.018**	**0.005**	**0.230**	**0.006**	**0.237**	**0.212**	**0.088**	**0.003**
**OTC4**	**0.076**	**0.050**	**0.043**	**0.029**	**0.016**	**0.017**	**0.046**	**0.002**
**NANOG**	**0.012**	**0.013**	**0.046**	**0.048**	**0.025**	**0.018**	**0.164**	**0.001**
